# Genetic factors underlying boron toxicity tolerance in rice: genome-wide association study and transcriptomic analysis

**DOI:** 10.1093/jxb/erw423

**Published:** 2016-11-18

**Authors:** Joao Braga de Abreu Neto, María Clara Hurtado-Perez, Monika A. Wimmer, Michael Frei

**Affiliations:** 1 Abiotic Stress Tolerance in Crops, INRES, University of Bonn, Bonn, Germany

**Keywords:** Abiotic stress, cereals, genome mapping, mineral toxicity, oxidative stress, transcriptomics.

## Abstract

Boron (B) toxicity is a nutritional disorder affecting crop production in many parts of the world. This study explored genetic factors associated with B tolerance in rice (*Oryza sativa* L.) through an integrated genome mapping and transcriptomic approach. Variation in B tolerance was first evaluated by screening a panel of 137 *indica* genotypes in B toxic conditions (+2 mM B), followed by genome-wide association study (GWAS). Leaf bronzing and greenness were significantly correlated with shoot and root dry weight, but B uptake was not correlated with any stress phenotype. Single nucleotide polymorphism (SNP) markers exceeding a significance value of –log_10_ *P*>4.0 were obtained for four traits, namely leaf bronzing, shoot dry weight, root dry weight, and root length. Linkage disequilibrium block analysis of the corresponding chromosomal regions revealed candidate loci containing 75 gene models. Two contrasting genotypes from the panel were selected for transcriptomic analysis, which included gene ontology enrichment analysis of differentially regulated genes and investigating transcriptional responses of GWAS candidate genes. Characteristic expression patterns associated with tolerance or sensitivity were seen in genes related to biochemical binding, transport, transcriptional regulation, and redox homeostasis. These results advance the understanding of genetic and physiological factors associated with B tolerance in rice.

## Highlight

Genetic factors associated with boron toxicity tolerance in rice were explored through an integrated approach combining genome-wide association study and transcriptomics.

## Introduction

Boron (B) is an essential micronutrient for crops, and its availability in the irrigation water or in the soil represents an important factor for crop production. In many parts of the world including Japan, China, the USA, and Brazil, natural B levels are insufficient for crop production since this element is present in the soil as boric acid, which is easily leached out by rainfall (Bolaños *et al.*, 2004). For this reason, B is frequently added as fertilizer in agricultural systems (Cayton, 1985). At the same time, excess B negatively affects crop production in many parts of the world. In arid or semi-arid countries, the main cause of B accumulation in agricultural soils is the evaporation of B-rich groundwater, which reaches the topsoil by capillary transport (Nable *et al.*, 1997). Also, B toxicity often occurs in soils intrinsically high in B, for example in Australia, West and Northern Africa, or as a result of irrigation with B-enriched water (Schnurbusch *et al.*, 2010*a*). In rice, B toxicity was first diagnosed in soils from coastal Taiwan (Ponnamperuma and Yuan, 1966), on the farm of the International Rice Research Institute (IRRI), and other locations in the Philippines (Cayton, 1985). It has been reported to cause significant rice grain yield losses in soils of volcanic origin and coastal areas (Cayton, 1985; Dobermann and Fairhurst, 2000).

B is a plant nutrient for which there is usually a relatively small window between deficient and toxic concentrations, both highly detrimental to crop yields. While B deficiency can be resolved by application of B-enriched fertilizers, toxicity is a more difficult problem to manage, and breeding of tolerant varieties is considered as the most promising approach (Reid *et al.*, 2004). Toxicity symptoms can be diverse, depending on the degree of B mobility within the plant (Brown *et al.*, 2002). In rice, B has low mobility, and consequently symptoms of toxicity include reduced growth of roots and shoots, chlorosis (associated with lower leaf chlorophyll contents and photosynthetic rates, Supplementary Fig. S1 at *JXB* online) and necrotic spots starting at the leaf tip and margins of mature leaves, resulting from the accumulation of excessive B transported through the transpiration stream (Wimmer *et al.*, 2015).

Tolerance to B toxicity varies between plant species (Ferreyra *et al.*, 1997). Many crop species belong to the family Poaceae (Gramineae), which generally contain lower B concentrations and are more susceptible to high B supply than dicotyledonous species (Brown *et al.*, 2002). The B requirements of graminaceous species are also lower than those of dicotyledonous species (Goldbach, 1997) and correlate positively with the pectin contents of the cell walls (Hu and Brown, 1994; Hu *et al.*, 1996). Additionally, B tolerance varies among cultivars of the same species (Nable, 1988). This genetic diversity in adaptation should offer the possibility of breeding adapted crops (Reid *et al.*, 2004) using genome mapping and marker-assisted breeding approaches.

Genome-wide association study (GWAS) is a mapping method that uses more genetically diverse populations than the bi-parental backcrossing approach traditionally implemented in mapping of quantitative trait loci (QTLs). This technique is especially advantageous in rice, since most rice varieties are homozygous, and, once a line is genotyped, the genetic data can be reused many times across different phenotypes and environments (Zhao *et al.*, 2011). For example, Zhao *et al.* (2011) mapped 44 100 SNPs (single nucleotide polymorphisms) across 413 diverse accessions of rice and phenotyped those for 34 traits, including straighthead susceptibility, blast resistance, flowering time in different locations, and many yield-related and quality traits. These genomic data were further used in studies targeting phosphorus utilization efficiency (Wissuwa *et al.*, 2015), seed dormancy (Magwa *et al.*, 2016), aluminium tolerance (Famoso *et al.*, 2011), iron toxicity (Matthus *et al.*, 2015), ozone tolerance (Ueda *et al.*, 2015), nematode resistance (Dimkpa *et al.*, 2016), and now, in the present study, tolerance to B toxicity.

Three experiments were carried out. The first experiment represented a preliminary screening of four rice varieties with different B concentrations. The aim was to establish a suitable experimental system and to find the most suitable B concentration that would induce differential stress responses in rice. The second experiment was a GWAS using 137 varieties to establish associations between phenotypes related to B toxicity and loci containing candidate genes. Lastly, varieties of similar genetic background that showed contrasting responses to B toxicity in the previous experiment were used in a transcriptome study to elucidate mechanisms of tolerance and sensitivity and to examine the expression patterns of candidate genes.

## Materials and methods

### Growth conditions

All experiments were carried out in greenhouses in Bonn, Germany. Plants were grown in hydroponic culture during 28–35 d after germination, with natural light supplemented with artificial lighting to ensure a minimum light intensity of 300 µmol m^–2^ s^–1^ illumination (PAR), and 30 °C/25 °C day (16 h)/night (8 h) temperature. Seeds were germinated for 3 d in the dark at 28 °C in distilled water. On the fourth day, the seedlings were transferred to meshes floating on a solution containing 0.5 mM CaCl_2_ and 0.01 mM FeCl_3_. On the 10th day of the experiment, the seedlings were transplanted into hydroponic containers of 60 liter volume. Each container had a perforated lid accommodating 40 plants, and PVC tubes were installed at the bottom of the lid to separate the roots of individual plants. The containers were filled with a modified Yoshida solution (Yoshida *et al.*, 1976), which had the following full-strength composition: N 2.86 mM, P 0.26 mM, K 0.82 mM, Ca 0.8 mM, Mg 1.3 mM, Mn 7.3 µM, Mo 0.4 µM, B 14.8 µM, Zn 0.16 µM, Cu 0.16 µM, and Fe 28.7 µM. During the first week, a half-strength solution was used, which was subsequently replaced weekly with full-strength solutions. The pH was adjusted twice a week to 5.5.

### Btox1: preliminary experiment

Four lines of *indica* rice were used in a preliminary experiment to establish a suitable B treatment to induce toxicity in common high yielding rice varieties (IR29, IR64, and IR74), compared with a presumably tolerant landrace (Pokkali). Twenty-four seedlings of each genotype were germinated and grown under the conditions described above, and were submitted to different concentrations of boric acid in the hydroponic medium on the 17th day after germination: control plants (15 µM B), +0.5 mM B, and +2 mM B. After 10 d of treatment, plants were harvested and phenotyped as described below.

### Btox2: GWAS

The seeds of the 137 varieties of rice (*Oryza sativa* L. ssp. *indica*) used in this experiment were originally obtained from the IRRI. All varieties belonged to the *indica* group and they originated from 38 different countries. The different subpopulations were represented as follows: 73 IND (*indica*), 51 *Aus*, and 13 ADMIX (admixed). Details of the origin of each accession are shown in Supplementary Table S1. Genotype data representing 44 100 SNP markers were available for this population (www.ricediversity.org; Zhao *et al.*, 2011).

Each accession was replicated four times in both treatments in independent hydroponic containers, leading to a total of 28 containers (14 in each treatment with 40 plants each). The distribution of seedlings inside each treatment group was randomized and the seedling trays were moved to different positions inside the greenhouse twice a week during both seedling preparation and stress treatment to avoid effects of plant positions on growth.

Eight traits were observed in this experiment on the 10th day of treatment: greenness (SPAD value) and leaf bronzing score (LBS), tiller number (TN), shoot and root length, root and shoot DW, and shoot B concentration (SBC). For each variety, reported values are means of three or four biological replicate samples.

### Btox3: microarray experiment

Based on the results of the extensive screening conducted in the previous experiment, four accessions were chosen having a similar genetic background and similar growth under control conditions, but showing different degrees of tolerance to B toxicity. The two contrasting pairs were SML 242 (NSFTV. ID: 299, from Suriname, B tolerant) and MTU9 (NSFTV. ID: 109, India, B sensitive) (kinship 0.89); and the Chinese varieties Guan-Yin-Tsan (NSFTV. ID: 61, B tolerant) and Kun-Min-Tsieh-Hunan (NSFTV. ID: 97, B sensitive) (kinship 1.18). After 4 weeks, 24 plants of each variety were exposed to two different conditions (control and +2 mM B treatment), and subdivided into three biological replicates, each containing eight seedlings. One of these contrasting pairs, SML 242 and MTU9, were chosen for microarray analyses.

After 5 d of treatment, four seedlings of each variety/condition/biological replicate were harvested and stored in liquid nitrogen for RNA extraction, while the remaining plants were used for additional phenotyping such as DW determination and B analysis.

### Plant phenotyping

Necrotic spots were quantified using a modified LBS (Wu *et al.*, 2014), varying from 1 (no visible damage) to 5 (~80% or more of the leaf surface damaged) (Supplementary Fig. S1). In each plant, the three youngest fully expanded leaves were scored.

Chlorosis was quantified by measuring leaf greenness using a chlorophyll meter (SPAD 502, Konica Minolta, Osaka, Japan). For each plant, the second youngest fully expanded leaf was chosen and measured in three equally distributed points, starting 10 cm from the tip, along the blade of the selected leaf. The leaf greenness value given for each plant was an average of these three readings.

Tiller number and shoot and root length were determined at harvest, and shoot and root DW were measured after oven-drying samples for at least 72 h at 70 °C.

B concentration was determined in whole shoots in the Btox2 experiment and separately in leaf blades, stems, and roots in the Btox3 experiment. Samples of 50 mg of dried and finely ground material were pressure digested in 2 ml of concentrated nitric acid for 6 h at 180 °C and diluted with Milli-Q water to a maximum of 10 ml. All samples were analyzed in triplicate. The determination of B followed a miniaturized spectrophotometric curcumin method (Wimmer and Goldbach, 1999) with some modifications: the concentration of the curcumin solution was reduced (curcumin/methyl-isobutyl-ketone 25 mg/50 ml) and the solution was not filtered. Phase separation was accelerated by a brief centrifugation step (2 min, 10 000 rpm). The final measurement at λ=550 nm was done using resistant microplates (Brandplates lipograde™ ref.781 842) and a NanoQuant Infinite M200 PRO microplate reader (Tecan, Männedorf, Switzerland). A standard curve with references ranging from 0 to 1 µg B ml^–1^ was used for quantification.

### Association mapping

In order to associate the phenotypic traits observed in the Btox2 experiment with genetic markers, a GWAS was conducted using TASSEL 3.0. software (Bradbury *et al.*, 2007). Nine phenotypic traits were used: LBS, relative (treated:control) values of leaf greenness, root length (RL), root dry weight (RDW), tiller number (TN), shoot length (SL), and shoot dry weight (SDW) in addition to the absolute SBC in the control and treated plants. Values that were >3 SDs from the averages were considered as outliers and excluded from the following analysis. The association was based on the SNP markers, kinship matrix, and principal component analysis (PCA) matrix according to Zhao *et al.* (2011). This SNP array covers all 12 chromosomes of the rice genome, offering around one SNP every 10 kb. As recommended by Brachi *et al.* (2011), SNPs that showed a minor allele frequency of <5% were removed, leaving 26 863 polymorphic markers for the subsequent analysis. A mixed linear model (MLM) was applied (P3D for variance component analysis and compression level set to optimum level), including both PCA and kinship data. Manhattan plots were displayed, and the threshold for significantly associated markers was set to *P*<0.0001 (i.e. –log_10_ *P*>4.0), as previously suggested (Zhao *et al.*, 2011; Matthus *et al.*, 2015; Ueda *et al.*, 2015).

To identify genes associated with the significant markers, a linkage disequilibrium (LD) analysis was conducted using Haploview 4.2 (Barrett *et al.*, 2005). Heat-maps were displayed showing the LD blocks according to Gabriel *et al.* (2002), where the upper 95% confidence bounds of the LD (D' value) exceeded 0.98 and the lower bounds exceeded 0.70. LD blocks determined the regions that included the significant SNPs, which were taken as candidate loci. Then, using the positions from the Rice Genome Annotation Project MSU7 database (Rice Genome Browser: http://rice.plantbiology.msu.edu), the annotations of all gene models in the linkage blocks were obtained.

The phenotypic data were also analyzed using two-factorial ANOVA, comparing the variation between treated and control plants and between the different genotypes for each observed trait (*P*<0.001). The correlation between traits was computed by Pearson’s correlation coefficient.

### RNA extraction and microarray slide hybridization

For RNA extraction and transcriptomic analyses, plants from the accessions MTU9 and SML 242 from the experiment Btox3 were used. Shoot samples were ground in liquid N_2_ and total RNA was isolated from ~100 mg of shoot samples using a plant RNA extraction kit (Peqlab, Erlangen, Germany). Genomic DNA was removed using on-column RNase-free DNase (Peqlab).

RNA samples were first analyzed regarding purity (Nanodrop 2000c, Thermo Fisher Scientific Inc., Wilmington, DE, USA) and integrity (Bioanalyzer, Agilent Technologies, Santa Clara, CA, USA). Only samples with RNA integrity number >8 were used for microarray slide hybridization (OakLabs GmbH, Hennigsdorf, Germany). The cRNA for one-color microarray slide hybridization was generated from 50 ng of RNA. The 8 × 60K microarray slides (Agilent 60-mer SurePrint technology, Agilent Technologies) contained 59 336 *indica* rice-specific probes that were designed based on the *indica* rice genome database from the Beijing Genome Institute (BGI) (http://rice.genomics.org.cn). Since gene annotations are not available from the BGI database, all probe sequences were first mapped to coding sequences of *japonica* rice from the MSU database (http://rice.plantbiology.msu.edu) using MUMmer 3.0 (Kurtz *et al.*, 2004), then probe sequences were blasted against the BGI database in the RISE platform (Rice Information System, http://rise2.genomics.org.cn/page/rice/index.jsp) to obtain the gene ID and Gene Ontology (GO) information.

### Microarray statistical analysis and sequence comparison

Raw fluorescence intensities were processed using the Bioconductor packages LIMMA (Smyth, 2005); background corrections were made with the robust multiarray average (RMA) method and quantile normalization. The ArrayQualityMetrics package (Kauffmann and Huber, 2013) was used to assess the quality of the normalized data sets. Seven contrasts were tested with LIMMA: the treatment effect (T) was evaluated for each genotype and for the average of both genotypes; the genotypic effect (G) was evaluated comparing the genotypes in control and B treatment and for the average of both treatments; and the interaction of treatment and genotype was tested. After fitting the data into a linear model, the standard errors were corrected using a simple empirical Bayes model. Moderated *t*-statistic and log-odds of differential expression [log_2_FC (fold change)] were computed for each contrast for each probe. Values associated with a false discovery rate (FDR) >0.05 (FDR=5%) were excluded. Subsequently, the probes were matched to their loci in the MSU Rice Genome Annotation Project Release 7 (Kawahara *et al.*, 2013). Probes with no match or ambiguously matching multiple loci were discarded. In the case of multiple probes representing the same locus, the average of those probes was used. Genes with log_2_FC≥1 were considered up-regulated and those with log_2_FC≤ –1 down-regulated in response to the treatment. GO enrichment analysis of differentially expressed genes (DEGs) was conducted using the agriGO analysis toolkit, with the rice gramene locus data set as reference (Du *et al.*, 2010). Genes contained in loci identified by GWAS, and showing differential regulation in the tolerant but not in the intolerant genotype, were nominated for further sequence analysis. Coding sequences and 2 kb upstream sequences for a subset of 21 accessions were downloaded from the public data repositories oryzaSNP (Alexandrow *et al.*, 2015) and TASUKE (Kumagai *et al.*, 2013) and aligned using MEGA7 software (Kumar *et al.*, 2016). *t*-tests were performed to investigate the effect of each polymorphic SNP on phenotypic traits.

### Quantitative RT-PCR

A 300 ng aliquot of total RNA was reverse transcribed with the GoScript™ Reverse Transcription System (Promega, Mannheim, Germany) and quantitative PCR (qPCR) was performed using the GoTaq^®^ qPCR master mix (Promega) processed in the StepOne Plus Realtime PCR System (AB Biosystems, Darmstadt, Germany). Eleven genes were analyzed using primers described in Supplementary Table S6, all with efficiency >80% as shown by serial dilutions of cDNA templates. Expression data were quantified using the comparative ∆∆CT method (Schmittgen and Livak, 2008) with the expression level of the sensitive line in the control treatment as calibrator and splicing factor U2AF gene (LOC_Os05g48960) as the endogenous reference (Höller *et al.*, 2014). Relative expression values from qRT-PCR were then plotted against those obtained by the microarray hybridization (after normalizing by setting the expression level of SML 242 in the control as 1, and log_2_ transformation of these relative values) and the correlation coefficient (*R* ^2^) was determined.

## Results

### Rice growth and B toxicity symptoms

In order to establish a suitable screening system and to determine phenotypic traits for the subsequent GWAS, four different varieties of rice were tested under three B concentrations (experiment Btox1). Chlorosis and necrotic spots were visible in some plants after 5 d of exposure, and after 10 d plants of all varieties showed symptoms. Although plants showed some chlorosis and necrotic spots with +0.5 mM B, these were not as evident as those observed at +2 mM B (Supplementary Fig. S2). Interestingly, some plants grown with +0.5 mM B showed an improvement in biomass over control and +2 mM B-treated plants, perhaps indicating a growth-stimulating effect of temporary and moderate B stress for some rice varieties (Supplementary Fig. S2).

In the subsequent extensive screening experiment (Btox2), the stress response of 137 varieties of rice at +2 mM B was monitored. All phenotypic traits showed a statistically significant difference between B stress (for 10 d) and control (Table 1). Detrimental effects included decreased growth (i.e. reduction in tiller number, root and shoot length, and root and shoot DW). Chlorosis was reflected in reduced leaf greenness. Necrotic lesions were not observed in control plants (average LBS=1), but were evident in plants from all tested varieties (average LBS=2.3) in the B stress treatment. Although in the treated condition the amount of B added to the medium was 100 times higher than in the control solution, the average increase observed in SBC was only 13-fold (Table 1).

**Table 1.** Effect of boron treatment on mean phenotypic values of 137 different accessions of rice

	Control	B treated	Treatment	Genotype	*Aus* versus IND
Leaf greenness (SPAD value)	34.3 (0.3)	28.2 (0.3)	***	***	***
Leaf bronzing score	1.0 (0.00)	2.2 (0.04)	***	NS	***
Root length (cm)	21.0 (0.3)	16.6 (0.3)	***	***	***
Root dry weight (mg)	75.7 (2.7)	48.1 (1.8)	***	NS	***
Tiller number	3.0 (0.1)	1.8 (0.1)	***	***	***
Shoot length (cm)	57.8 (0.9)	50.6 (0.9)	***	***	NS
Shoot dry weight (mg)	363.7 (13.4)	225.1 (8.1)	***	***	***
Shoot B concentration (µg g^–1^)	43.9 (1.4)	577.4 (8.4)	***	NS	NS

Data are from experiment Btox2, where plants were exposed to control conditions or +2 mM B for 10 d.

The mean phenotypic values of control and B-treated plants (*n*=137) are shown, with the SEM in parentheses.

ANOVA tests were performed with treatment, genotype, and subpopulations (*Aus* vs. IND) as fixed effects. ***P<0.001. NS, not significant.

Correlations between the phenotypic traits were tested by determining Pearson’s *r* (Table 2). As expected, the highest positive correlations were observed between the biomass (SDW and RDW) and growth traits such as SL, RL, and TN. Leaf greenness (SPAD value) was also positively correlated with RDW, SDW, and TN, indicating that a lack of chlorophyll was related to biomass losses. Consistently, LBS was negatively correlated with TN and SDW, further corroborating the associations between symptom formation, growth, and biomass. Somewhat surprisingly, no significant correlation was observed between SBC in the stress treatment and any of the other phenotypic traits. The only significant correlations occurred between SBC in the control and LBS (positive) and between SBC in the control and B stress treatment (negative).

**Table 2.** Pearson’s correlation coefficients of the phenotypic traits measured in 137 different rice accessions

	Relative SPAD	LBS	Relative RL	Relative RDW	Relative TN	Relative SL	Relative SDW	SBC Control
LBS	–0.08							
Relative RL	–0.13	–0.06						
Relative RDW	0.26	–0.14	–0.03					
Relative TN	0.26	–0.22	–0.03	0.47				
Relative SL	0.16	–0.06	–0.14	0.38	0.16			
Relative SDW	0.29	–0.17	0.01	0.79	0.56	0.43		
SBC Control	–0.02	0.27	–0.09	0.00	0.00	0.08	0.06	
SBC B Treated	0.13	–0.09	0.13	0.02	0.10	–0.08	0.01	–0.19

Data are from experiment Btox2, where plants were exposed to control conditions or +2 mM B for 10 d.

LBS, leaf bronzing score; RL, root length; RDW, root dry weight; TN, tiller number; SL, shoot length; SDW, shoot dry weight; SBC, shoot boron concentration.

Shaded values are significant at *P*<0.05, *P*<0.01, or *P*<0.001, darker shading indicates higher significance.

**Fig. 1.**
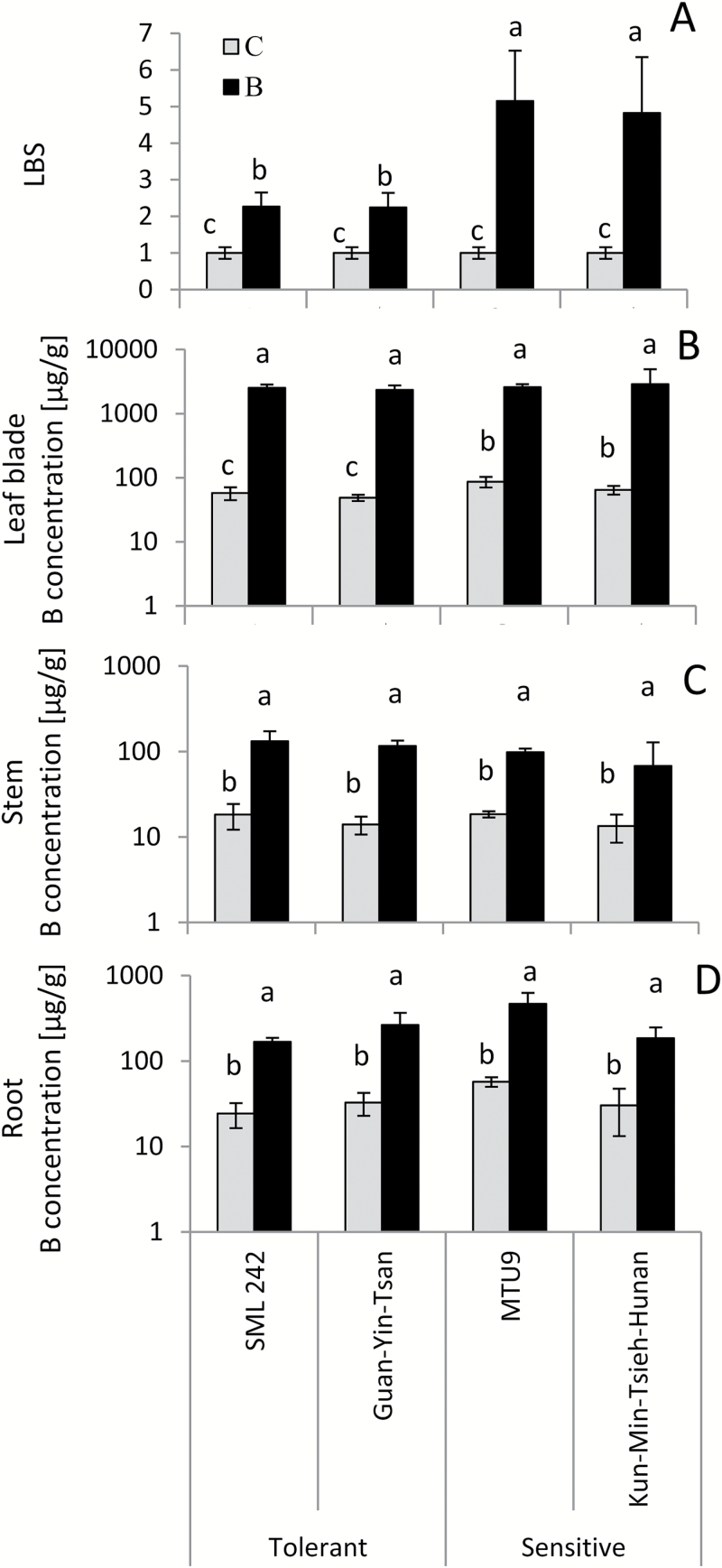
Phenotypic responses of four contrasting rice varieties to B stress. Data are from experiment Btox3, where seedlings of four rice varieties (B-tolerant SML242 and Guan-Yin-Tsan; B-sensitive MTU9 and Kun-Min-Tsieh-Hunan) were subjected to control conditions (C, gray bars) and +2 mM B (B, black bars). (A) Leaf bronzing score (LBS); (B) B concentration in leaf blades; (C) B concentrations in stem and leaf sheaths; (D) B concentration in roots. Data bars show mean values and SEs; bars not sharing the same letter in the same variety are significantly different at *P*<0.05 by Student’s *t*-test.

In the third experiment (Btox3), four contrasting genotypes were selected based on the traits determined in Btox2 for in-depth analysis of transcriptional responses to B toxicity. Two tolerant lines (SML 242 and Guan-Yin-Tsan) and two rather sensitive ones (MTU9 and Kun-Min-Tsieh-Hunan) were tested. The response in terms of LBS was consistent with the tolerance ranking obtained in Btox2 (Fig. 1). Sensitive and tolerant lines did not differ in B concentrations in any plant part in the B stress treatment, but the sensitive genotypes had higher B concentrations compared with tolerant genotypes in leaf blades in the control (Fig. 1).

### Association mapping

We conducted association mapping to identify loci underlying the genetic regulation of the phenotypic traits. Association mapping for each trait included the determination of SNPs exceeding a significance value of −log_10_ *P*>4.0 (an arbitrary threshold that had been previously applied by others, e.g. Ueda *et al.*, 2015; Wissuwa *et al.*, 2015), analyses of LD blocks surrounding each highly significant (−log_10_ *P*>4.0) SNP, and curation of a list of candidate genes contained within these blocks (Supplementary Table S3). Out of 26 863 markers included in this analysis, we identified 18 SNP markers that satisfied the established threshold (Supplementary Table S2). For many of the measured traits, phenotype–genotype associations were rather weak, as demonstrated by *P*-values for the most significant markers that barely deviated from the null distribution [see quantile–quantile plots (q–q plots) in Supplementary Figs S3.1B–3.7]. The trait LBS yielded three markers exceeding −log_10_ *P*>4.0, but the phenotypic data were not normally distributed (Supplementary Fig. S3.1). Therefore, in the following section, the mapping results for two phenotypes will be discussed in detail, namely relative SDW and relative RL, as these traits were normally distributed and yielded SNPs exceeding −log_10_ *P*>4.0.

### Relative shoot dry weight

**Fig. 2.**
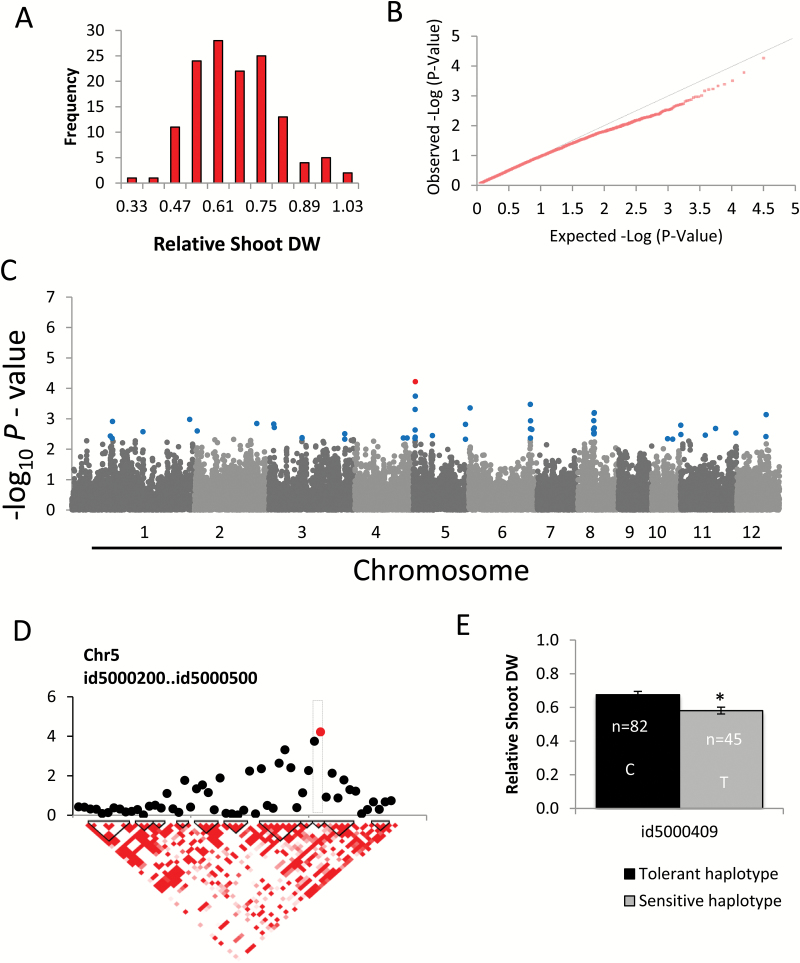
Association mapping result for relative shoot dry weight (SDW). (A) Frequency distribution of observed relative shoot length. (B) q–q plot of expected and observed *P*-values. (C) Manhattan plots from association mapping using the MLM. The top 50 SNPs are shown in blue and the SNPs exceeding the significance threshold of *P*<0.0001 are shown in red. (D) Detailed view of the peak region on chromosome 5. Pair-wise linkage disequilibrium (LD) between SNP markers is indicated as D' values: dark red indicates a value of 1 and white indicates 0. The dotted squares denote the LD blocks that contain significant SNPs. (E) Mean phenotypic values of tolerant and sensitive haplotypes for the highly significantly (−log_10_ *P*>4.0) associated SNP markers; bars represent mean values of relative SDW and SEs, *n*=number of lines possessing each allele. Asterisks indicate a significant difference (*P*<0.05) between haplotype groups.

This trait showed substantial phenotypic variation in the population and was close to normally distributed (Fig. 2A). The q–q plot suggested that the MLM did not overestimate significances, but the most significant SNPs also did not exceed the null distribution (Fig. 2B). The highest peak with an SNP marker (id5000409) exceeding −log_10_ *P*>4.0 was observed on chromosome 5 (Fig. 2C), and was located in an LD block containing only two markers (Fig. 2D). When the population was grouped into two allele variants for the significant SNP id5000409, a significant difference in relative SDW between these two haplotypes was observed (Fig. 2E).

### Relative root length

**Fig. 3.**
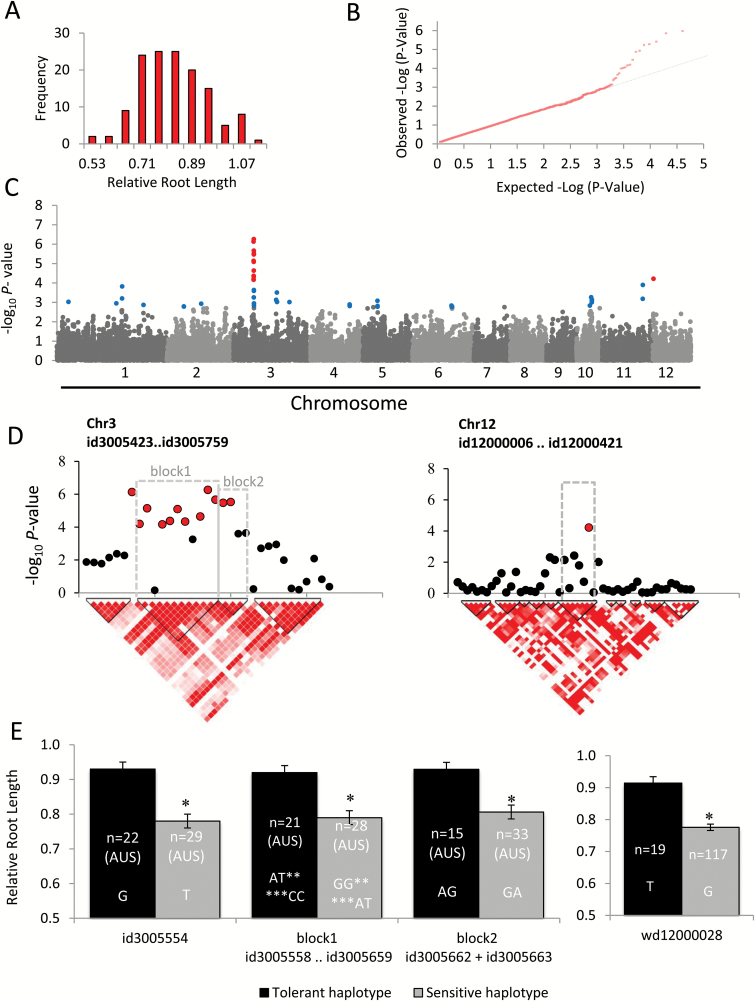
Association mapping result for relative root length (RL). (A) Frequency distribution of observed relative root length. (B) q–q plot of expected and observed *P*-values. (C) Manhattan plots from association mapping using the MLM. The top 50 SNPs are shown in blue and the SNPs exceeding the significance threshold of *P*<0.0001 are shown in red. (D) Detailed view of the peak regions on chromosomes 3 and 12. Pair-wise linkage disequilibrium (LD) between SNP markers is indicated as D' values: dark red indicates a value of 1 and white indicates 0. The dotted squares denote the LD blocks that contain significant SNPs. (E) Mean phenotypic values of tolerant and sensitive haplotypes for the highly significantly (−log_10_ *P*>4.0) associated SNP markers; bars represent mean values of relative RL and SEs, *n*=number of lines possessing each allele. When polymorphisms are only observed in one subpopulation this is indicated. Asterisks indicate a significant difference (*P*<0.05) between haplotype groups.

Relative RL yielded the most robust mapping results as the phenotype data were normally distributed (Fig. 3A), the q–q plots demonstrated strong deviation of the most significant SNPs from the null distribution (Fig. 3B), and a large number of SNPs located in a clearly defined linkage block were significantly associated with this trait (Fig. 3C, D). Twelve SNPs located within a segment of 222.28 kb on chromosome 3 exceeded the significance threshold, and five of those SNPs were located in coding regions of the genes LOC_Os03g19020 (PHD-finger family protein), LOC_Os03g19080 (unknown expressed protein), and LOC_Os03g19280 (argininosuccinate lyase). Another significant marker for this trait (wd12000028) was localized on chromosome 12 in an LD block containing eight genes, including DNA cytosine methyltransferase Zmet3 (LOC_Os12g01800), a cation/H^+^ exchanger ATCHX (LOC_Os12g01820), and two pentatricopeptides (LOC_Os12g01850 and LOC_Os12g01910) (Supplementary Table S3). In total, the LD blocks defined as candidate loci contained 42 genes.

### Genes differentially expressed in response to high B

The transcriptomic response to high B stress was analyzed by microarray hybridization in two accessions that showed consistent stress responses in both experiments Btox2 and Btox3. The tolerant SML 242 (originating from Surinam) and the sensitive MTU9 (originating from India) are both from the IND subpopulation of the Asian rice. They were chosen because they had similar morphology in the control treatment but differed significantly in their response to high B. Based on the lack of correlation between shoot B concentrations and stress phenotypes in Btox2, and similar shoot B concentration in both genotypes in different plant compartments after B stress treatment (Fig. 1), we assumed that contrasting tolerance was due to shoot-based mechanisms rather than root-based exclusion of B or root-to-shoot transport mechanisms. Therefore, we focused our analysis on shoot transcriptomics. For confirmation of microarray expression data, qRT-PCR analyses were performed for 11 genes (Supplementary Table S6), which were in good agreement (*R* ^2^=0.80) (Supplementary Figs S4, S5).

**Fig. 4.**
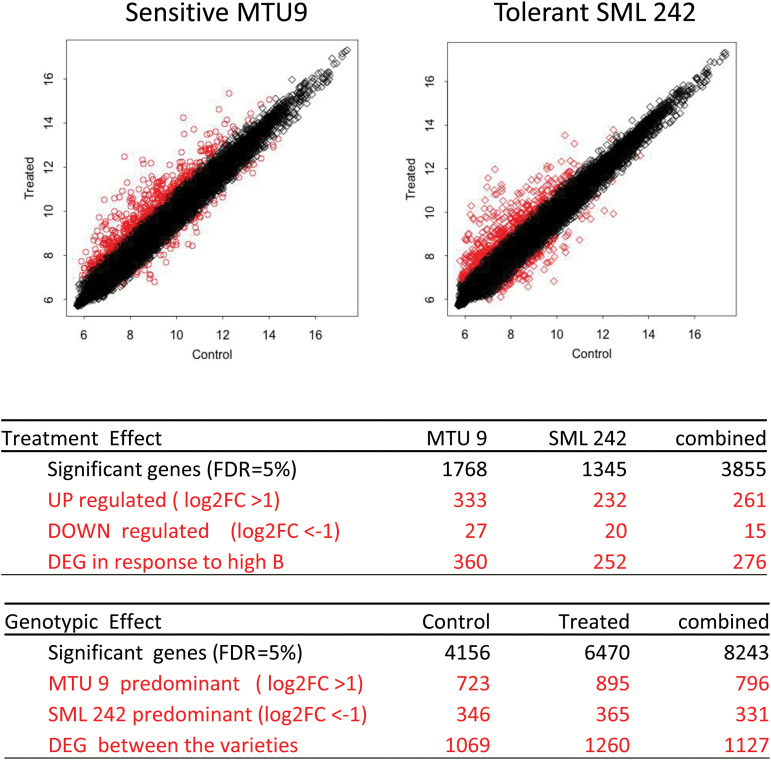
Summary of microarray analysis of two different rice varieties, MTU9 (sensitive) and SML242 (tolerant) in response to B stress treatment. Data are from experiment Btox3. Differentially expressed genes (DEGs) are shown in red.

**Fig. 5.**
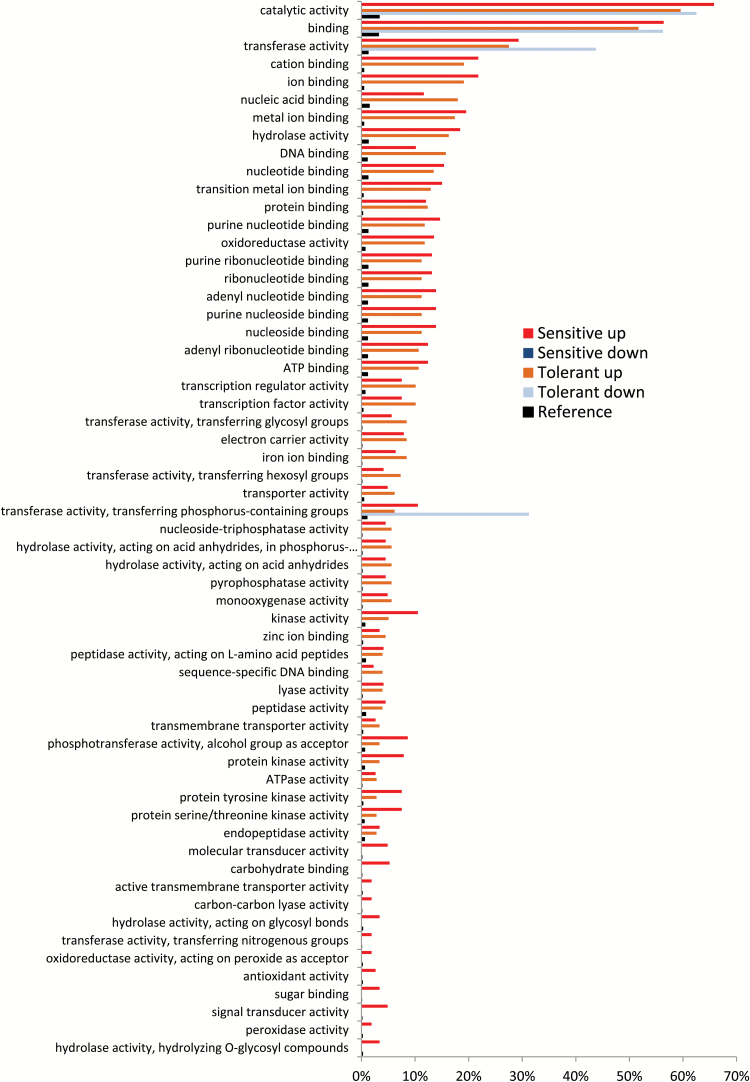
Gene ontology (GO) enrichment analysis ‘molecular function’ of differentially expressed genes (DEGs) in two contrasting rice genotypes MTU9 (sensitive) and SML242 (tolerant) in response to boron toxicity. Data are from experiment Btox3. Analysis was computed by AgriGO using the Rice Gramene locus data set as reference. Only significantly enriched terms (FDR=5%) are shown. (This figure is available in colour at *JXB* online.)

In the transcriptomic analysis, contrasts for genotype and treatment effects were first calculated. In further analysis, we considered only significant probe values (FDR=5%) (Supplementary Table S4), and genes that showed a difference of at least |log_2_FC|≥1 between the contrasting conditions. In total, 1127 genes were differentially expressed between the two varieties across both treatments. Interestingly, more genes were responsive in the stress condition (1260) than in the control (1069), and the sensitive genotype exhibited a larger number of DEGs than the tolerant genotype, suggesting that many transcriptional responses represented stress responses rather than tolerance mechanisms (Fig. 4; Supplementary Table S5). GO analysis (Fig. 5; Supplementary Table S7) was conducted for DEGs in both genotypes in order to establish response profiles, which are characteristic of tolerance versus sensitivity. Catalytic activity, transferase activity, and various types of ‘binding’ were the dominant B-responsive GO terms in the category ‘molecular function’. GO terms, in which the tolerant genotype was more responsive than the sensitive genotype, included processes related to transcriptional and post-transcriptional regulation, such as nucleic acid binding, transcription regulator activity, and transcription factor activity. In contrast, GO terms related to typical stress response pathways such as antioxidant activity or signal transducer activity were more highly or exclusively up-regulated in the sensitive genotype (Fig. 5).

**Fig. 6.**
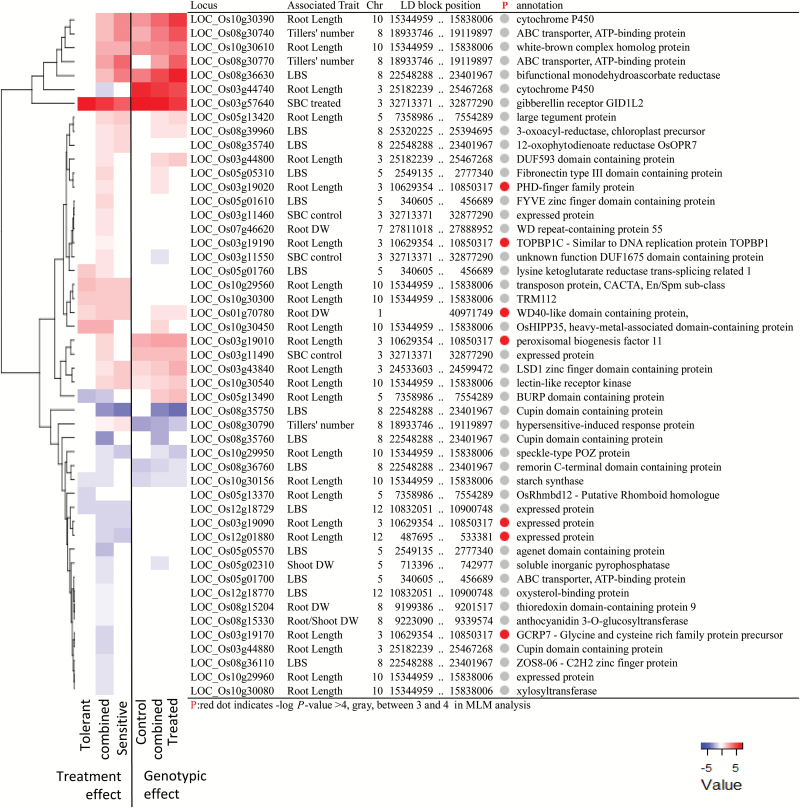
Relative expression of 49 differentially regulated genes, which were located within candidate loci from the genome-wide association study. Data are from experiment Btox3. Lines: SML242 (tolerant), MTU9 (sensitive), or the average of both (combined values). Genes chosen by the association of observed traits with SNP markers. Treatment effect was expressed as the expression value of treated relative to control samples, while genotype effect was expressed as the expression value of the sensitive relative to the tolerant genotype. (This figure is available in colour at *JXB* online.)

GWAS and transcriptome analysis were integrated by constructing a heat map summarizing the expression data for all DEGs, which were simultaneously nominated as candidate genes by GWAS (Fig. 6). In this analysis we included all genes contained in LD blocks surrounding SNP markers with a −log_10_ *P*>3.0 rather than −log_10_ *P*>4.0 to increase the number of genes included in the analysis (Supplementary Table S3). The resulting gene list was largely composed of regulatory genes involved in signal transduction, transcription, and post-transcriptional modification (Fig. 6), as well as uncharacterized proteins. Four genes that were differentially regulated in the tolerant but not in the intolerant line were subjected to sequence comparison based on a subset of 21 accessions from the original mapping population (Supplementary Tables S8–S11). Those four genes contained a number of polymorphic SNPs in the coding sequences and 2 kb upstream regions that significantly affected phenotypic traits based on *t*-tests.

## Discussion

### Symptom formation, growth, and biomass are correlated but are not determined by shoot B concentrations in rice

In our preliminary experiment, we tested different B concentrations, namely +0.5 mM and +2 mM B, regarding their effectiveness in inducing B toxicity symptoms in rice grown in hydroponic culture. Both concentrations were above the threshold considered to cause B toxicity in rice when occurring in soil solution (>2.5 mg l^–1^, equivalent to >0.23 mM; Dobermann and Fairhurst, 2000), and were lower than the concentration reported to induce a 10% yield reduction in rather tolerant rice varieties (120 mg l^–1^, equivalent to 11.1 mM; Cayton, 1985). In the GWAS, we opted for a +2 mM B treatment as this concentration caused significant responses in all measured phenotypes within 10 d (Table 1), and also emphasized genotypic differences as evidenced by near normal distribution of most phenotypic data except for LBS (Figs 2, 3; Supplementary Figs S3.1–3.7). Many previous hydroponic experiments with other species employed higher concentrations. For example, Jefferies *et al.* (1999) applied a B stress treatment of 100 mg l^–1^ (equivalent to 9.25 mM) in their experiments with barley, and Schnurbusch *et al.* (2007) and Pallotta *et al.* (2014) applied stress treatments up to 10 mM in their experiments with wheat. In the present study, the lowest B treatment was chosen that still induced severe but non-lethal toxicity in a wide range of varieties.

The symptoms observed after 10 d of B treatment included reductions in growth and biomass, chlorosis, and necrosis (Table 1). The visual symptoms—chlorosis and necrosis—were significantly correlated with several growth and biomass parameters, indicating that symptom formation can serve as a reliable indicator for growth reductions due to B toxicity in rice. In contrast, shoot B concentrations, especially in the B toxicity treatment, were not correlated with symptom formation and biomass reductions (Table 2), and root B exclusion did not determine tolerance. This is at first surprising, as the scientific literature often suggests that reduced B accumulation is a predominant mechanism in B tolerance. For example, the ability to maintain low tissue B concentrations despite toxic B levels in the growth media was associated with tolerance in wheat (Paull *et al.*, 1991) and barley (Sutton *et al.*, 2007). Moreover, tolerance to high B levels in barley (Hayes and Reid, 2004), wheat (Reid, 2007), and Arabidopsis (Miwa *et al.*, 2007) was associated with high expression of root B efflux transporters, which also indicated the importance of B exclusion at the root. Other studies, however, did not find a correlation between shoot B content and tolerance level. For example, a major QTL for B tolerance in barley was correlated with leaf symptom scores, but not with shoot B concentrations (Jefferies *et al.*, 1999). In agreement with our results, Ochiai *et al.* (2008) also observed that contrasting rice varieties did not differ significantly in terms of shoot B concentrations, and suggested that differences in cellular tolerance might contribute to B tolerance in rice. A possible mechanism of such cellular tolerance was demonstrated by Reid and Fitzpatrick (2009) for tolerant varieties of wheat and barley expressing B efflux transporters in leaves, which pump B from the symplast into the less sensitive apoplast. However, no efflux transporters were differentially regulated in leaves in our microarray analysis (Supplementary Table S5), indicating that other mechanisms, such as regulatory effects of transcription factors as suggested by Nozawa *et al.* (2006) (and described in detail below), were more likely to be involved in B tolerance in the present study. Therefore, our next aim was to identify candidate genes and mechanisms that could be associated with shoot-based tolerance to B toxicity.

### Most robust phenotype–genotype associations were seen in root length

To our knowledge, this was the first study genetically dissecting B tolerance in any crop species by GWAS. Compared with QTL mapping using bi-parental populations, GWAS harnesses broader genetic diversity, and potentially has higher mapping resolution, which can facilitate the identification of candidate genes in a single experiment (Huang and Han, 2014; Yano *et al.*, 2016). Previously, QTLs for B toxicity tolerance were reported for wheat (Schnurbusch *et al.*, 2007) and barley (Emebiri *et al.*, 2009; McDonald *et al.*, 2010). In their study with rice, Ochiai *et al.* (2008) determined one major QTL on chromosome 4, which affected relative shoot length. In our GWAS, we did not observe any significant peak for this trait on chromosome 4. There are two possible explanations for this lack of co-localization: (i) the previously reported QTLs were mapped in a population of bi-parental crosses of Nekken-1 (tolerant *japonica*) and IR36 (sensitive *indica*), two varieties which were absent from our diversity panel; or (ii) the previous study used much higher B concentrations (60 mg l^–1^, equivalent to 5.6 mM) and younger seedlings (7 d old).

In barley, a major QTL conferring B tolerance was mapped on chromosome 4 (Jefferies *et al.*, 1999), and the underlying gene was the B efflux transporter Bot1, a BOR1 ortholog (Sutton *et al.*, 2007). Similarly, root-specific B transporters were characterized as the causative genes underlying major QTLs for B tolerance in wheat (Schnurbusch *et al.*, 2007; Pallotta *et al.*, 2014). In contrast, B uptake and shoot concentrations were not correlated with the stress phenotypes in the present experiment, which is in line with the fact that no specific B transporters were among the genes contained within the loci identified by GWAS.

The majority of SNP markers exceeding the significance threshold of −log_10_ *P*>4.0 were associated with relative RL. This trait has been used in various previous mapping experiments investigating B tolerance in crops. Interestingly, major QTLs associated with RL were reported in barley (Jefferies *et al.*, 1999) and wheat (Schnurbusch *et al.*, 2007), and affected important agronomic traits. Another aspect that makes this trait interesting in our study is that the tolerant haplotypes (i.e. accessions carrying favorable allele combinations at significantly associated SNPs) represented the minor alleles (Fig. 3E), suggesting that there is scope for improving B tolerance in the majority of genotypes by replacing sensitive alleles.

### Transcriptional responses to B toxicity in contrasting genotypes reveal characteristic sensitive and tolerant expression profiles

An additional aim of our study was to characterize physiological responses associated with shoot tolerance to B toxicity by investigating transcriptional regulation in contrasting genotypes. GO enrichment analysis of DEGs suggested that many of the B-responsive genes were associated with transcriptional regulation and biochemical binding. This is plausible because B plays an important role in transcriptional regulation and readily binds to a large number of molecules due to its capacity to form complexes with diols and polyols, and could be involved in cross-linking of molecules (Brown *et al.*, 2002; Bassil *et al.*, 2004; Bolaños *et al.*, 2004; Reid, 2010). In fact it has been suggested that B toxicity causes metabolic disturbances by complexing with various molecules including NAD^+^ and ribose moieties of RNA (Reid, 2010; Marschner, 2012). In addition, a number of ribosomal proteins and transcription factors from Arabidopsis conferring B tolerance in yeast have been identified (Nozawa *et al.*, 2006). One possible explanation is that transcription factors may hinder the binding of B to *cis*-hydroxyls on ribose molecules that are exposed during gene splicing and in tRNA, which could result in disturbance of transcription and/or translation (Nozawa *et al.*, 2006).

Some GO categories were more responsive in the tolerant and others in the sensitive genotype (Fig. 5). Twelve GO categories were exclusively enriched in the sensitive genotype, including signal transduction and antioxidant activity, which represent important stress responses to a broad range of abiotic stresses (de Abreu Neto and Frei, 2016). Conceivably these pathways represented secondary responses to stress in the sensitive genotype, while tolerance to high B was related rather to the GO categories over-represented in the tolerant genotype. These included regulatory pathways such as transcriptional regulation, transferase activity, and nucleic acid/DNA binding. Remarkably, ‘transferase activity, transferring phosphorus containing groups’ was over-represented among the down-regulated genes in the tolerant, but not in the sensitive genotype. This might indicate that tolerance mechanisms require some phosphorylation/de-phosphorylation activity.

### GWAS candidate loci contain differentially expressed genes with possible involvement in B tolerance

Integration of GWAS and transcriptomics can help to gain understanding of biological mechanisms underlying loci that affect target traits (Conde *et al.*, 2013). Therefore, we investigated the transcriptional regulation of all genes contained in the LD blocks identified as candidate loci through GWAS (Fig. 6). Many of the DEGs contained in these loci were involved in three processes possibly involved in B tolerance: transport, transcription, and redox homeostasis (Fig. 6).

Borate exporters (BORs) were fist described in a high B-requiring Arabidopsis mutant (Noguchi *et al.*, 1997). Two types of BORs are known: those that facilitate B transport under low B conditions and those involved in B exclusion for high B tolerance (Wakuta *et al.*, 2015). Previous studies indicated the importance of those pumps in cereals such as barley (Reid, 2007; Sutton *et al.*, 2007; Reid and Fitzpatrick, 2009), wheat (Reid, 2007), and rice (Miwa and Fujiwara, 2011). However, the transport of this element is not exclusive to BORs, as several members of the major intrinsic protein (MIP) superfamily also have transport function (Schnurbusch *et al.*, 2010*b*; Hanaoka *et al.*, 2014; Kumar *et al.*, 2014; Mosa *et al.*, 2016). Our list of candidate DEGs contained no specific B transporters but several ATP-binding cassette (ABC) transporters that were responsive in the sensitive genotype (LOC_Os08g30740 and LOC_Os08g30770) or down-regulated in both genotypes (LOC_Os05g01700). Similarly, ABC transporters were also highlighted as DEGs in a microarray study of barley in a B toxicity treatment (Öz *et al.*, 2009).

Transcription factors are involved in the regulation of all biological processes, including B stress responses. Notably the suppression of the NAC-like transcription factor BET-1 (Boron Excess Tolerant 1) was shown to increase the B tolerance of rice plants (Ochiai *et al.*, 2011), and two Arabidopsis MYB transcription factors (AtMYB13 and AtMYB69) were shown to enhance B tolerance in yeast without significantly reducing B concentrations (Nozawa *et al.*, 2006). Different zinc-finger transcription factors were also highlighted in Arabidopsis (Kasajima and Fujiwara, 2007) and barley microarray studies (Öz *et al.*, 2009). Several DEGs nominated in this study, such as the PHD-finger family protein (LOC_Os03g19020), the MYB family transcription factor (LOC_Os03g19120), or the WD40-like domain-containing protein (LOC_Os01g70780), putatively function in transcriptional regulation, and could be involved in B stress response or tolerance.

Cellular redox homeostasis is involved in the response to many abiotic stresses (de Abreu Neto and Frei, 2016), including B toxicity (Hamurcu *et al.*, 2013; Landi *et al.*, 2014). Several of the candidate DEGs (Fig. 6) are involved in redox homeostasis, including the bi-functional monodehydroascorbate reductase (LOC_Os08g36630), which forms part of the antioxidant network of the ascorbate glutathione cycle (Noctor and Foyer, 1998), or OsORP7 (LOC_08g35740), regulating the plant hormone jasmonic acid (Tani *et al.*, 2008), which in turn mediates oxidative cell death processes (Kangasjärvi *et al.*, 2005). Consistent with the observations from the GO enrichment analysis (Fig. 5), these two oxidative stress-related genes were induced exclusively in the sensitive genotype, indicating that their up-regulation represents a typical sensitive response.

Based on the expression patterns, genes that were differentially regulated in the tolerant but not in the sensitive genotype can be considered as interesting candidates involved in tolerance. Four genes fulfilled this criterion: (i) lysine ketoglutarate reductase trans-splicing related 1 (LOC_Os05g01760), which could be involved in transcriptional programs regulating plant amino acid metabolism in response to abiotic stress (Less and Galili, 2008); (ii) OsHIPP35 (LOC_Os10g30450), which forms part of the family of heavy metal-associated isoprenylated plant proteins involved in heavy metal detoxification and transcriptional responses (de Abreu-Neto *et al.*, 2013); (iii) a BURP domain-containing protein (LOC_Os05g13490), which forms part of a largely uncharacterized gene family with multiple putative roles in rice including stress response (Ding *et al.*, 2009); and (iv) a starch synthase (LOC_Os10g30156), which is involved in the foliar build-up of storage carbohydrates (Lloyd and Kossmann, 2015). Based on transcriptional regulation, their putative functions, and polymorphisms, which seemed to affect boron toxicity tolerance in rice (Supplementary Tables S9–S11), these genes appear as interesting candidates for further investigations.

## Conclusions

This study demonstrated substantial variations in tolerance to B toxicity in rice, which was genetically dissected by GWAS. Surprisingly, B tolerance indicators such as chlorosis and necrosis were not correlated with B uptake and translocation into shoots, suggesting that shoot-based tolerance mechanisms played an important role. These could be related to transcriptional and other downstream regulatory mechanisms as suggested by converging evidence from GWAS and transcriptomic analysis in contrasting genotypes. A number of candidate genes associated with these mechanisms were proposed that warrant further investigation.

## Supplementary data

Supplementary data are available at *JXB* online.

Table S1. Description and phenotypic means of all 137 rice accessions used in the genome-wide association study (experiment Btox2).

Table S2. Mixed linear model (MLM) analysis of SNP markers associated with nine traits observed in the experiment Btox32.

Table S3. Candidate genes selected by GWAS.

Table S4. Complete data set for microarray analysis in experiment Btox3.

Table S5. Genes differentially expressed in shoots of control and B-stressed plants in experiment Btox3.

Table S6. Primer pairs used in qPCR analysis.

Table S7. GO enrichment analysis of differentially expressed genes (DEGs) in microarray analysis (experiment Btox3) in the tolerant line SML 242 and the sensitive line MTU9.

Table S8. Haplotype analysis of polymorphic SNPs contained in gene LOC_Os05g01760.

Table S9. Haplotype analysis of polymorphic SNPs contained in gene LOC_Os05g13490.

Table S10. Haplotype analysis of polymorphic SNPs contained in gene LOC_Os10g30156.

Table S11. Haplotype analysis of polymorphic SNPs contained in gene LOC_Os10g30450.

Fig. S1. Photos of boron toxicity symptoms in rice leaves.

Fig. S2. Phenotypic responses of four different rice varieties to different levels of boron.

Fig. S3.1–3.7. Association mapping results.

Fig. S4. Relative expression of 109 genes that were elected by the GWAS approach and show a genotypic effect in transcriptional regulation.

Fig. S5. Relative quantification of 10 rice genes used for validation of microarray data by qPCR.

Fig. S6. Validation of microarray data by qPCR with 10 selected genes.

## Supplementary Material

supplementary_tables_S1_S11

supplementary_figures_S1_S6

## Abbreviations:

ADMIX: admixed subpopulation
BGI: Beijing Genome Institute
DEG: differentially expressed gene
FC: fold change
FDR: false discovery rate
GO: Gene Ontology
GWAS: genome-wide association study
IND: indica subpopulation
IRRI: International Rice Research Institute
LBS: leaf bronzing score
LD: linkage disequilibrium
MIP: major intrinsic protein
MLM: mixed linear model
NSFTV ID: National Science Foundation-‘Exploring the Genetic Basis of Transgressive Variation in Rice’
PCA: principal component analysis
q–q plot: quantile–quantile plot
QTL: quantitative trait locus
RDW: root dry weight
RL: root length
RMA: robust multiarray average
SBC: shoot boron concentration
SDW: shoot dry weight
SD: standard deviation
SE: standard error
SL: shoot length
SNP: single nucleotide polymorphism
TN: tiller number.
