# Supplementary material for: Genetic factors underlying boron toxicity tolerance in rice: genome-wide association study and transcriptomic analysis
Source: J Exp Bot. 2016 Nov 18;68(3):687–700. doi: 10.1093/jxb/erw423 (PMC5444448; doi:10.1093/jxb/erw423)
Supplement: supplementary_figures_S1_S6 [file erw423_suppl_supplementary_figures_S1_S6.pdf]

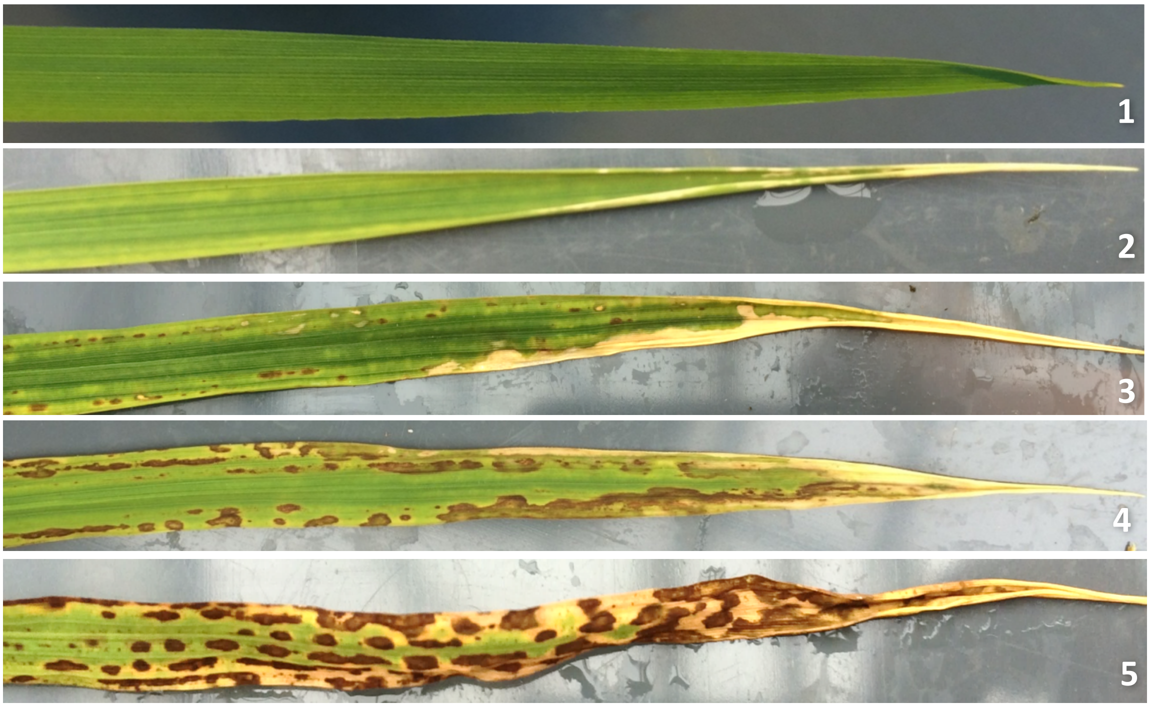

Supplementary Figure S1. Photos of boron toxicity symptoms in rice leaves. Leaf Bronzing Score (LBS) from a leaf without symptoms (1) to a leaf completely damaged (5) showing chlorosis and necrotic spots on the entire leaf surface.

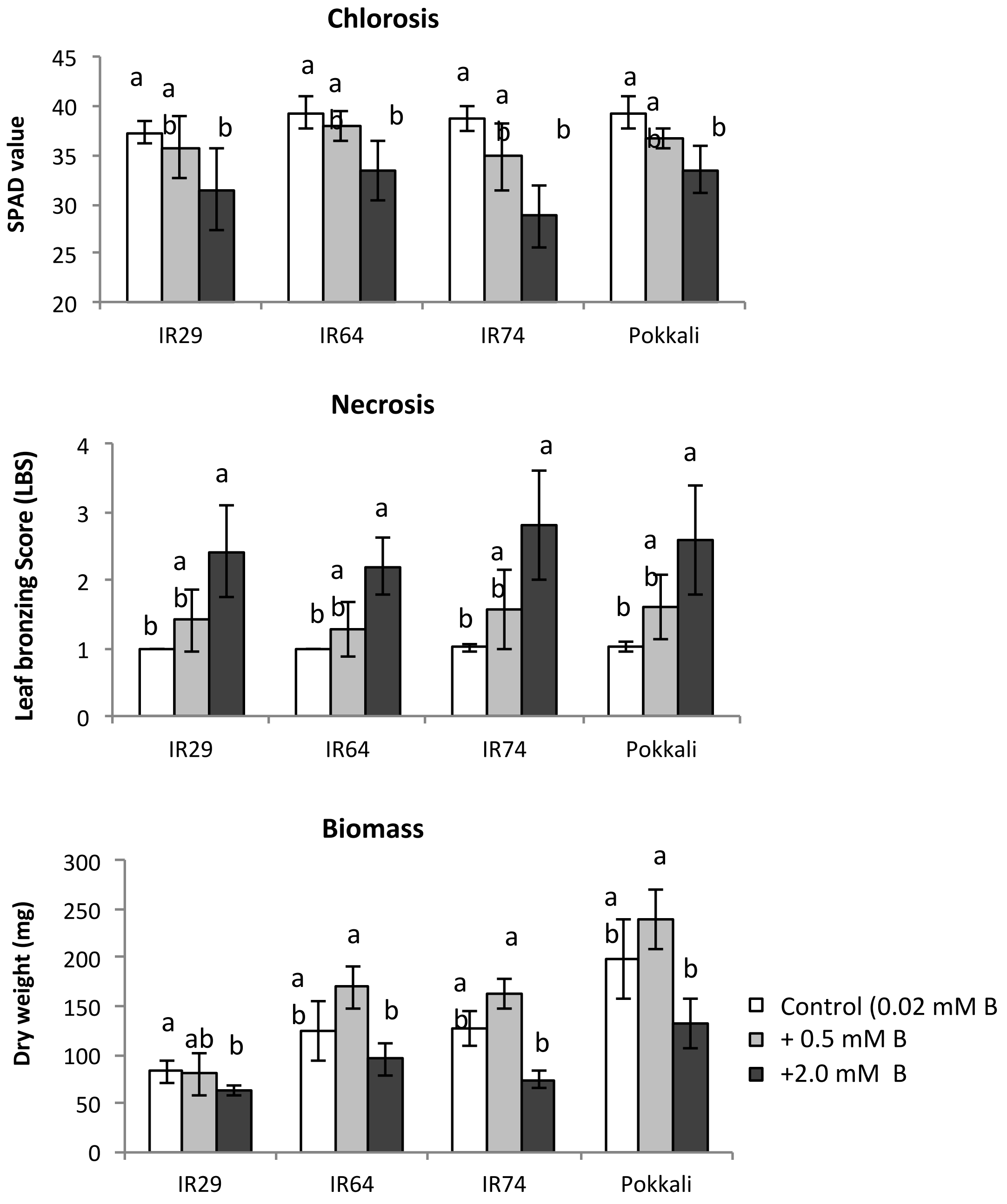

Supplementary Figure S2. Phenotypic responses of four different rice varieties to different levels of boron. Data are from experiment Btox1. Data bars show mean values and standard errors; bars not sharing the same letter in the same variety are significantly different at $P < 0.05$ by pairwise comparison.

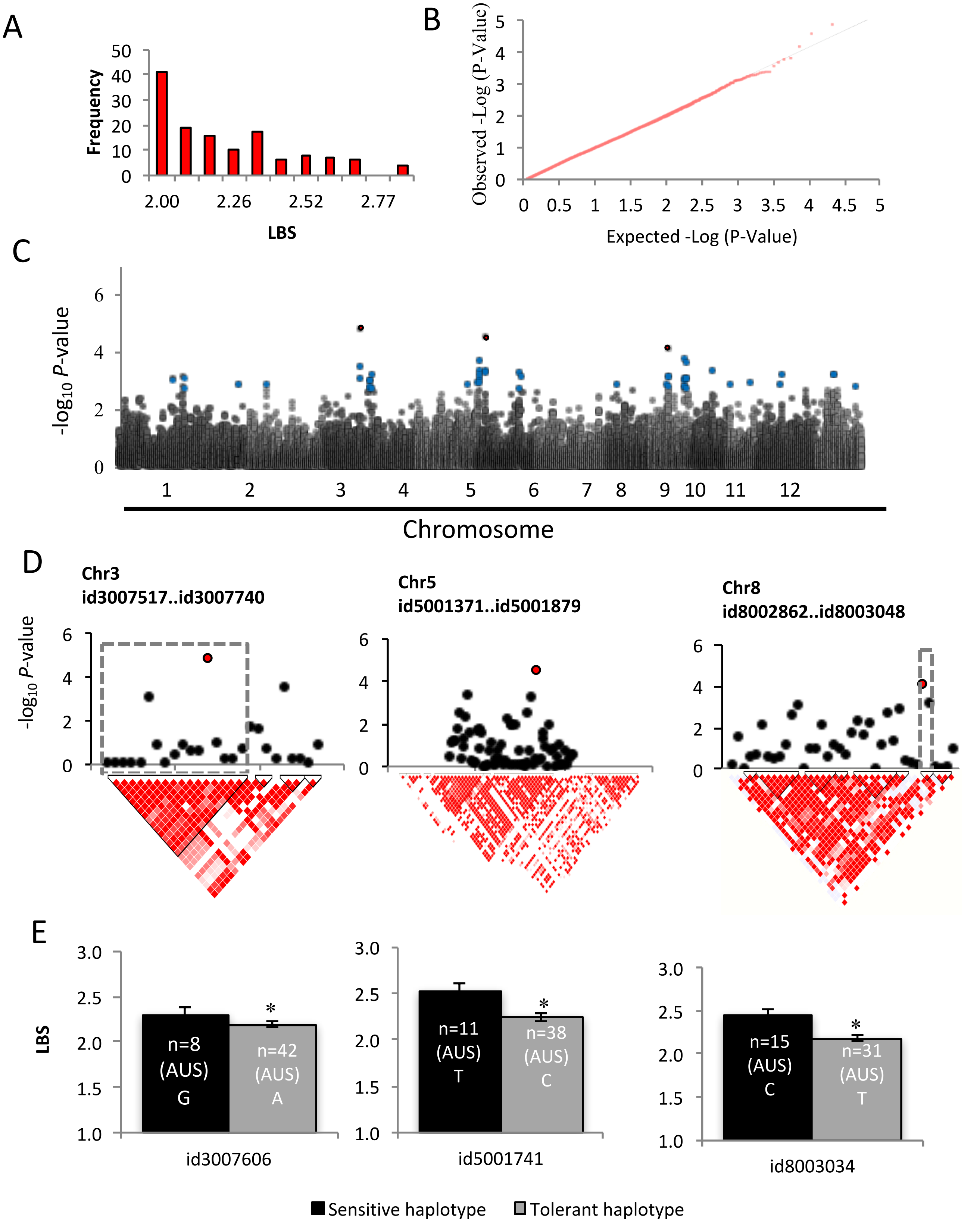

Supplementary Figure S3.1. Association mapping result for leaf bronzing score (LBS) (A) Frequency distribution of observed LBS. (B) q-q plot of expected and observed P values. (C) Manhattan plots from association mapping using the MLM. The top 50 SNPs are shown in blue and the SNPs exceeding the significance threshold of P<0.0001 are shown in red. (D) Detailed view of significant markers, pair-wise LD between SNP markers is indicated as D' values: dark red indicates a value of 1 and white indicates 0. The dotted squares in (D) denote the LD blocks that contain significant SNPs. (E) Tolerant and sensitive haplotypes of the highly significantly (−log10 P > 4.0) associated SNP marker, data bars show mean values of relative LBS and standard errors, n: number of lines possessing each allele. Asterisk indicates significant difference (P<0.05) between haplotype groups.

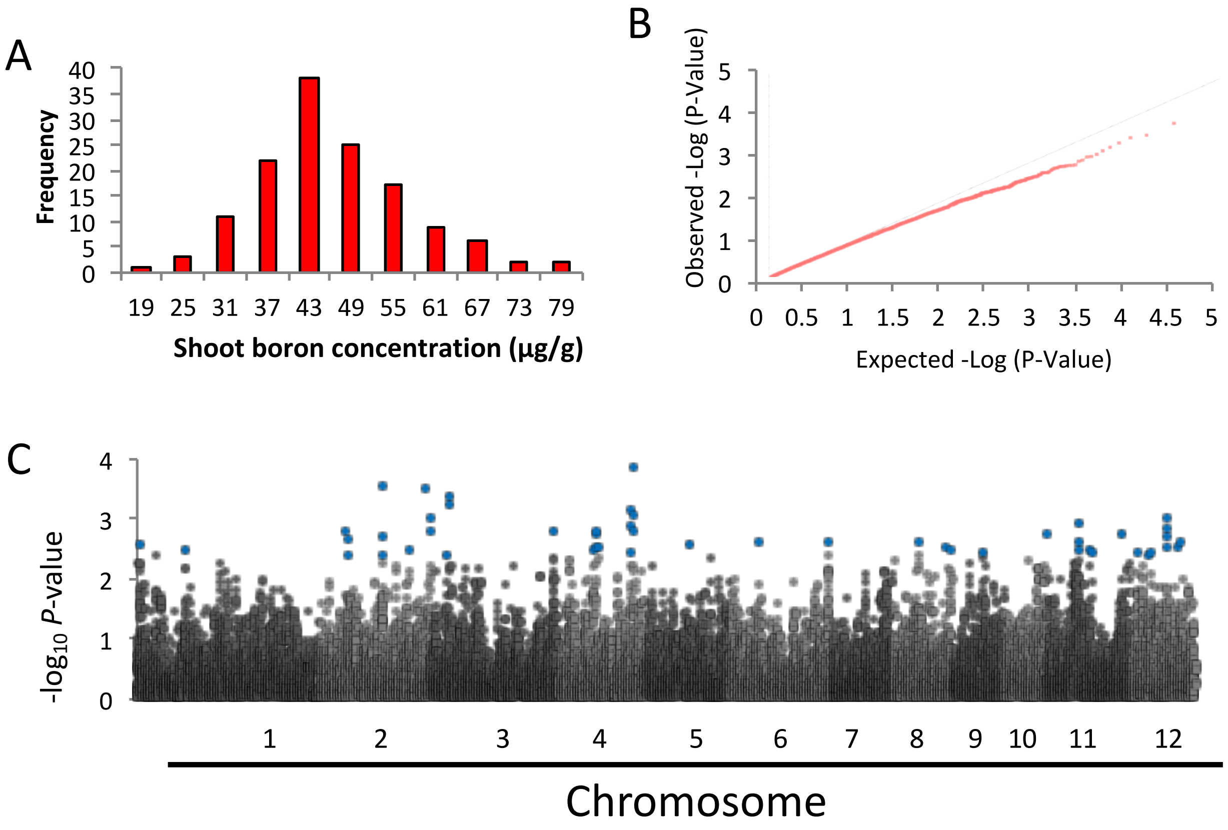

Supplementary Figure S3.2. Association mapping result for shoot boron concentration (SBC) in the control (A) Frequency distribution of SBC (B) q-q plot of expected and observed P values. (C) Manhattan plots from association mapping using the MLM. The top 50 SNPs are shown in blue.

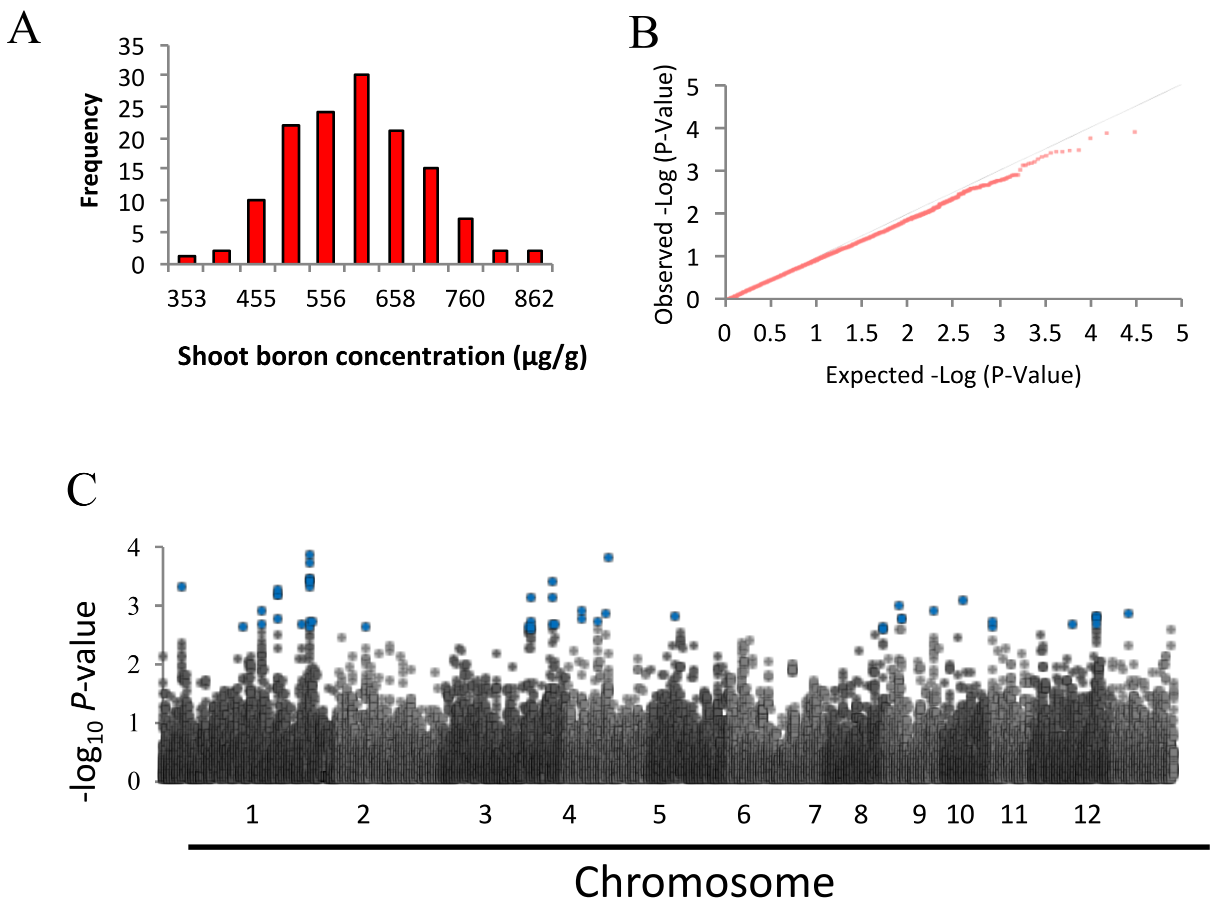

Supplementary Figure S3.3. Association mapping result for shoot boron concentration (SBC) in the boron stress treatment. (A) Frequency distribution of observed SBC. (B) q-q plot of expected and observed P values. (C) Manhattan plots from association mapping using the MLM. The top 50 SNPs are shown in blue.

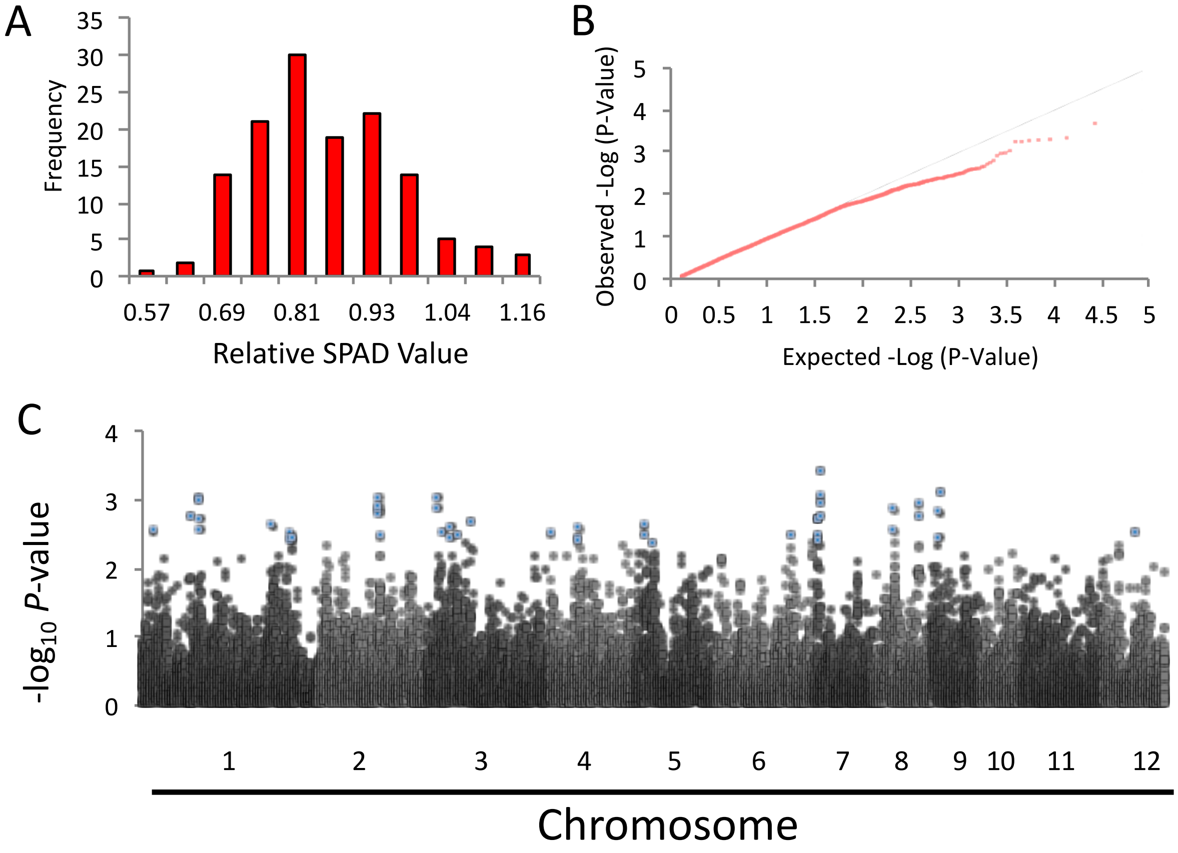

Supplementary Figure S3.4. Association mapping result for relative leaf greenness (SPAD values). (A) Frequency distribution of observed relative SPAD values. (B) q-q plot of expected and observed P values. (C) Manhattan plots from association mapping using the MLM. The top 50 SNPs are shown in blue.

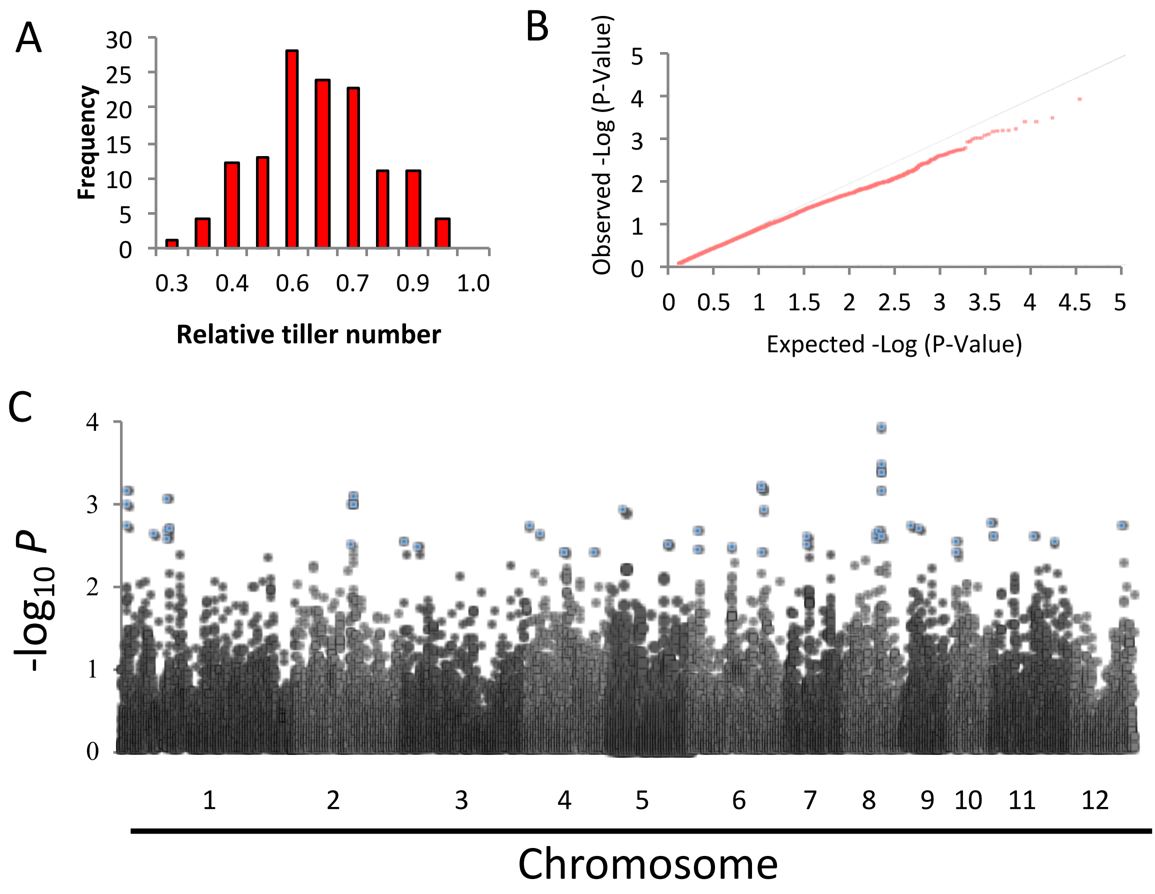

Supplementary Figure S3. Association mapping result for relative tiller number. (A) Frequency distribution of observed relative tiller number. (B) QQ plot of expected and observed P values. (C) Manhattan plots from association mapping using the MLM. The top 50 SNPs are shown in blue.

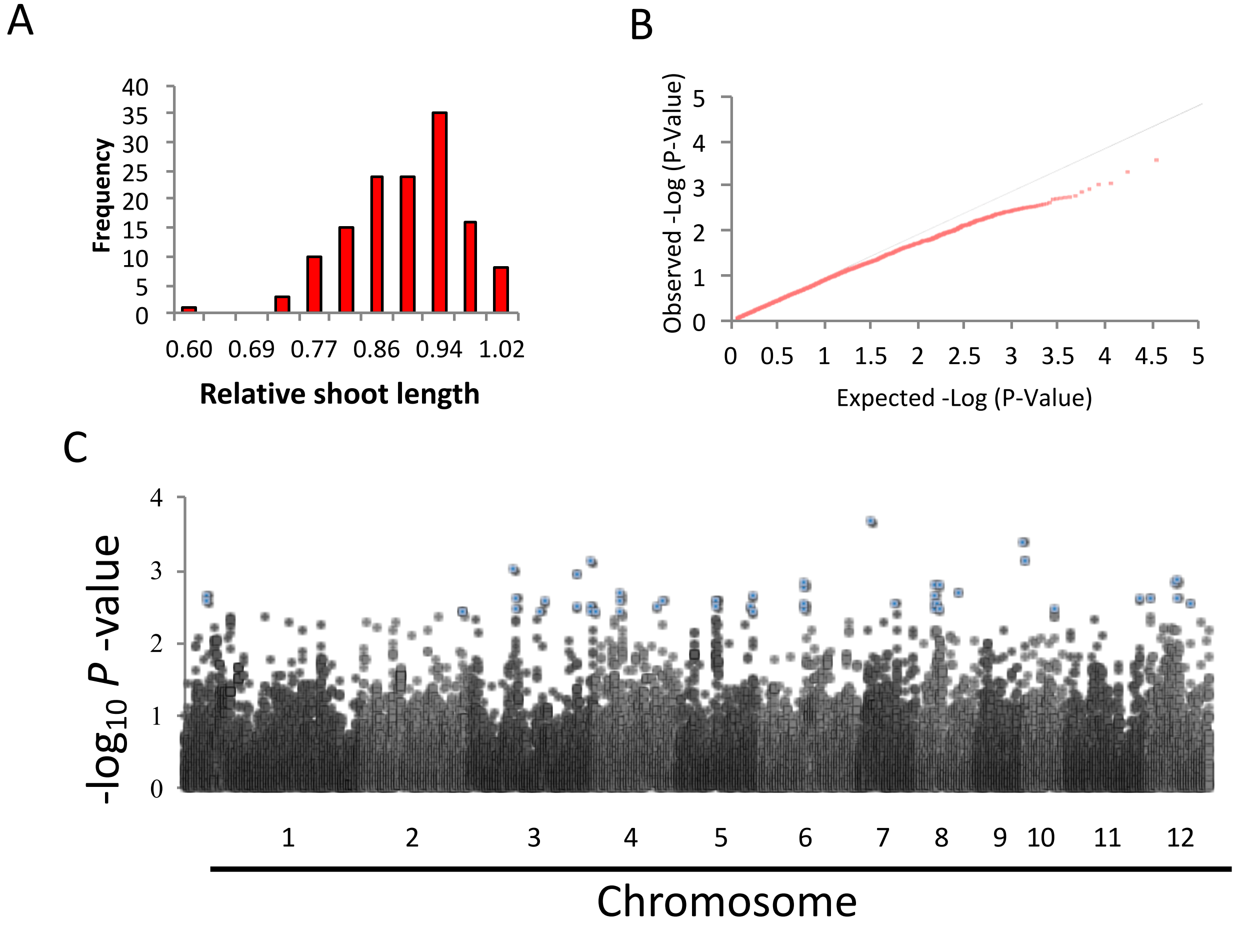

Supplementary Figure S3.6. Association mapping result for relative shoot length. (A) Frequency distribution of observed relative shoot length. (B) QQ plot of expected and observed P values. (C) Manhattan plots from association mapping using the MLM. The top 50 SNPs are shown in blue.

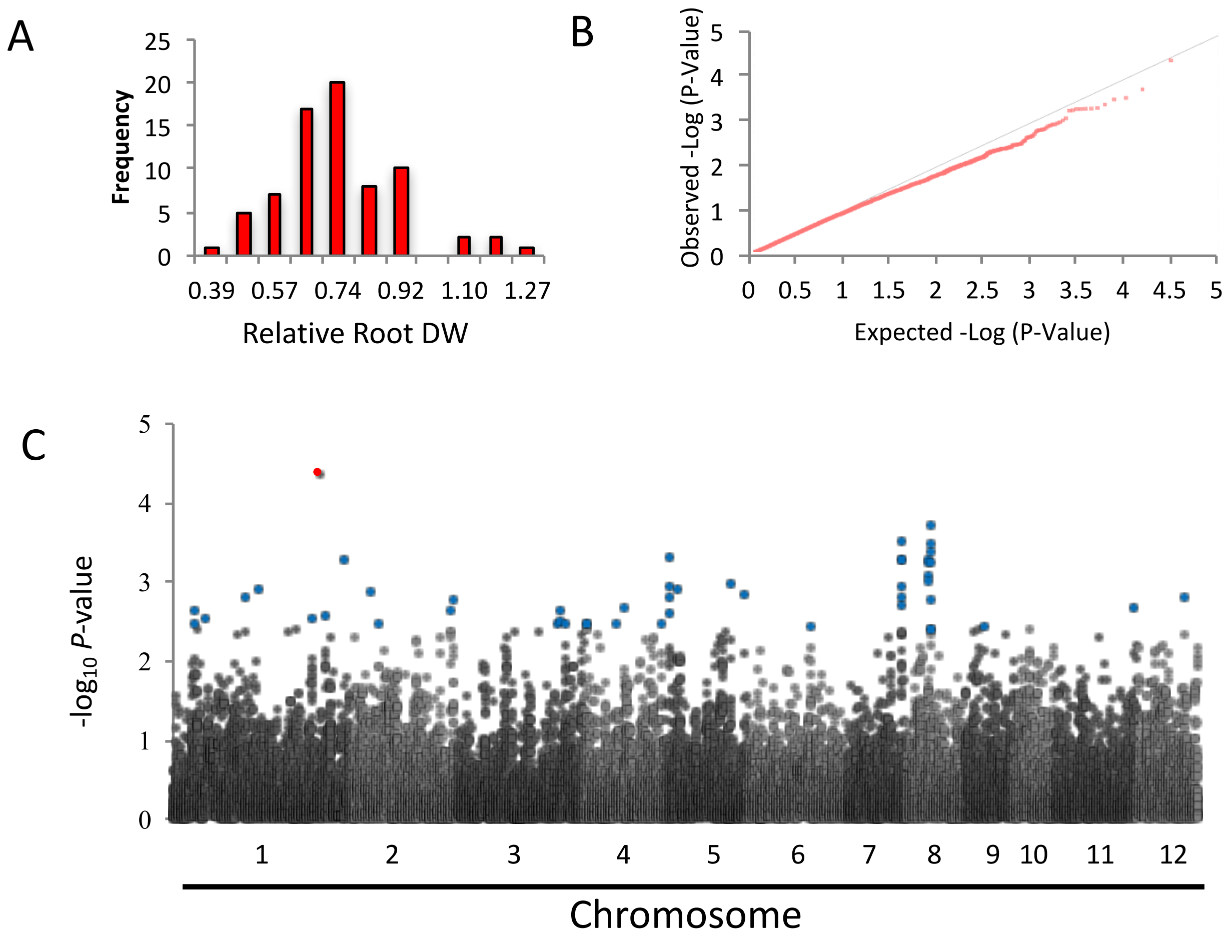

Supplementary Figure S3.7 . Association mapping result for relative root dry weight (DW). (A) Frequency distribution of observed relative root DW. (B) QQ plot of expected and observed P values. (C) Manhattan plots from association mapping using the MLM. The top 50 SNPs are shown in blue and the SNPs exceeding the significance threshold of P<0.0001 are shown in red.

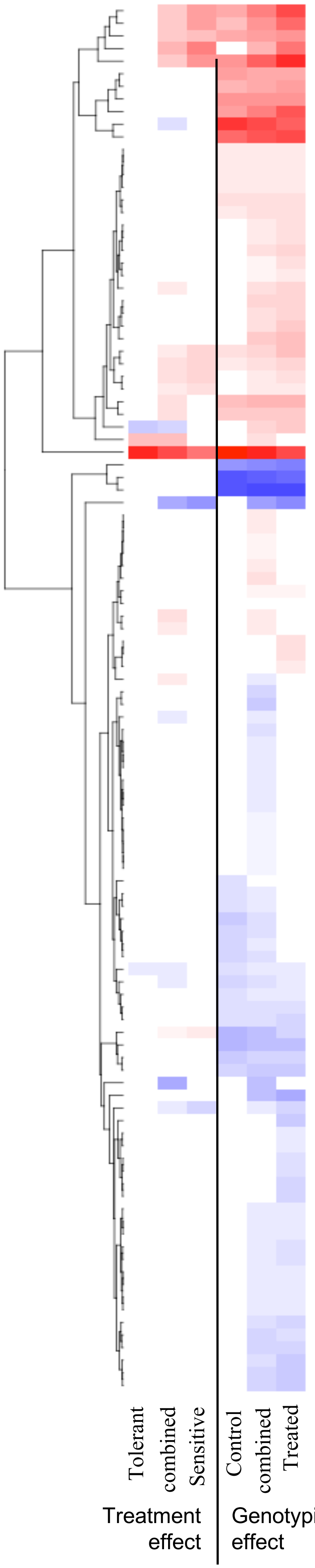

Locus	Associated Trait	Chr	LD block position	P	annotation
LOC_Os10g30390	Root Length	10	15344959 .. 15838006	gray	cytochrome P450
LOC_Os08g30740	Tillers' number	8	18933746 .. 19119897	gray	ABC transporter, ATP-binding protein
LOC_Os10g30610	Root Length	10	15344959 .. 15838006	gray	white-brown complex homolog protein
LOC_Os08g30770	Tillers' number	8	18933746 .. 19119897	gray	ABC transporter, ATP-binding protein
LOC_Os08g36630	LBS	8	22548288 .. 23401967	gray	bifunctional monodehydroascorbate reductase
LOC_Os05g13580	Root Length	5	7381676 .. 7554289	gray	OsCML18 - Calmodulin-related calcium sensor protein
LOC_Os03g26360	LBS	3	14988188 .. 15124843	red	expressed protein
LOC_Os03g57569	SBC Treated	3	32713371 .. 32877290	gray	expressed protein
LOC_Os08g36860	LBS	8	23117679 .. 23401967	gray	cytochrome P450
LOC_Os03g44740	Root Length	3	25182239 .. 25467268	gray	cytochrome P450
LOC_Os05g05390	LBS	5	2549135 .. 2777340	gray	expressed protein
LOC_Os10g30520	Root Length	10	15871614 .. 15934503	gray	ras-related protein
LOC_Os08g36420	LBS	8	22548288 .. 23043330	gray	metal cation transporter
LOC_Os10g30600	Root Length	10	15871614 .. 15934503	gray	tyrosine protein kinase domain containing protein
LOC_Os03g11470	SBC Control	3	5898875 .. 6007474	gray	DEAD/DEAH box helicase domain containing protein
LOC_Os04g52850	SBC Control	4	31476593 .. 31482643	gray	OTU-like cysteine protease family protein
LOC_Os05g05240	LBS	5	2549135 .. 2777340	gray	serine/threonine protein phosphatase
LOC_Os08g39970	LBS	8	25320225 .. 25394695	gray	ENT domain containing protein
LOC_Os08g35850	LBS	8	22548288 .. 23043330	gray	expressed protein
LOC_Os08g36790	LBS	8	22548288 .. 23401967	gray	bZIP transcription factor
LOC_Os08g35880	LBS	8	22548288 .. 23043330	gray	OsFBX294 - F-box domain containing protein
LOC_Os08g36840	LBS	8	22548288 .. 23401967	gray	glycoprotein 3-alpha-L-fucosyltransferase A
LOC_Os03g44800	Root Length	3	25182239 .. 25467268	gray	DUF593 domain containing protein
LOC_Os12g29330	SBC Control	12	17282862 .. 17416055	gray	no apical meristem protein
LOC_Os03g44780	Root Length	3	25182239 .. 25467268	gray	elongation factor Tu family protein
LOC_Os08g30719	Tillers' N	8	18933746 .. 19119897	gray	expressed protein
LOC_Os03g26460	LBS	3	14988188 .. 15124843	red	CS domain containing protein
LOC_Os03g43840	Root Length	3	24533603 .. 24599472	gray	LSD1 zinc finger domain containing protein
LOC_Os10g30540	Root Length	10	15344959 .. 15838006	gray	lectin-like receptor kinase
LOC_Os05g13420	Root Length	5	7358986 .. 7554289	gray	large tegument protein
LOC_Os08g39960	LBS	8	25320225 .. 25394695	gray	3-oxoacyl-reductase, chloroplast precursor
LOC_Os03g19010	Root Length	3	10629354 .. 10850317	red	peroxisomal biogenesis factor 11
LOC_Os03g11490	SBC control	3	32713371 32877290	gray	expressed protein
LOC_Os05g13490	Root Length	5	7358986 .. 7554289	gray	BURP domain containing protein
LOC_Os10g30450	Root Length	10	15344959 .. 15838006	gray	OsHIPP35, heavy-metal-associated domain-containing protein
LOC_Os03g57640	SBC Treated	3	32713371 .. 32877290	gray	gibberellin receptor GID1L2
LOC_Os03g57349	SBC Treated	3	32713371 .. 32877290	gray	expressed protein
LOC_Os03g57520	SBC Treated	3	32713371 .. 32877290	gray	VQ domain containing protein
LOC_Os03g26229	LBS	3	14988188 .. 15124843	red	40S ribosomal protein S9-1
LOC_Os08g35750	LBS	8	22548288 .. 23043330	gray	Cupin domain containing protein
LOC_Os05g05230	LBS	5	2549135 .. 2777340	gray	expressed protein
LOC_Os03g57430	SBC Treated	3	32713371 .. 32877290	gray	expressed protein
LOC_Os02g39410	SBC Control	2	23754247 .. 23781398	gray	protein phosphatase 2C
LOC_Os08g36774	LBS	8	22548288 .. 23401967	gray	zinc finger family protein
LOC_Os08g36994	LBS	8	22548288 .. 23401967	gray	protein transport protein
LOC_Os03g19260	Root Length	3	10629354 .. 10850317	red	expressed protein
LOC_Os05g05270	LBS	5	2549135 .. 2777340	gray	sucrose-phosphatase
LOC_Os03g26791	LBS	3	15292722 .. 15298374	gray	expressed protein
LOC_Os05g05310	LBS	5	2549135 .. 2777340	gray	Fibronectin type III domain containing protein
LOC_Os03g19020	Root Length	3	10629354 .. 10850317	red	PHD-finger family protein
LOC_Os10g29700	Root Length	10	15344959 .. 15838006	gray	expressed protein
LOC_Os03g57410	SBC Treated	3	32713371 .. 32877290	gray	RING-H2 finger protein ATL5D
LOC_Os07g46660	Root DW	7	27811018 .. 27888952	gray	ubiquitin carboxyl-terminal hydrolase domain containing protein
LOC_Os03g11550	SBC Control	3	32713371 .. 32877290	gray	unknown function DUF1675 domain containing protein
LOC_Os05g05460	LBS	5	2549135 .. 2777340	gray	EF hand family protein
LOC_Os03g26180	LBS	3	14988188 .. 15124843	red	expressed protein
LOC_Os05g02310	Shoot DW	5	713396 .. 742977	gray	soluble inorganic pyrophosphatase
LOC_Os05g05480	LBS	5	2549135 .. 2777340	gray	OsDegp6 - Putative Deg protease homologue
LOC_Os08g36010	LBS	8	22548288 .. 23043330	gray	OsFBX295 - F-box domain containing protein
LOC_Os08g36120	LBS	8	22548288 .. 23043330	gray	retrotransposon protein, putative, unclassified
LOC_Os03g11530	SBC Control	3	32713371 .. 32877290	gray	nucleotide pyrophosphatase/phosphodiesterase
LOC_Os03g44850	Root Length	3	25182239 .. 25467268	gray	expressed protein
LOC_Os03g43850	Root Length	3	24533603 .. 24599472	gray	recA protein
LOC_Os08g36000	LBS	8	22548288 .. 23043330	gray	OsFBL51 - F-box domain and LRR containing protein
LOC_Os10g30340	Root Length	10	15344959 .. 15838006	gray	expansin precursor
LOC_Os03g26380	LBS	3	14988188 .. 15124843	red	transposon protein, putative, unclassified
LOC_Os03g57450	SBC Treated	3	32713371 .. 32877290	gray	CAMK CAMK like.2 - CAMK includes calcium/calmodulin depedent protein kinases
LOC_Os08g40030	LBS	8	25320225 .. 25394695	gray	CUP-SHAPED COTYLEDON3
LOC_Os05g13340	Root Length	5	7358986 .. 7554289	gray	expressed protein
LOC_Os07g46630	Root DW	7	27811018 .. 27888952	gray	AMP deaminase
LOC_Os12g29350	SBC Control	12	17282862 .. 17416055	gray	ATP binding protein
LOC_Os08g15420	LBS	8	9380466 .. 9381057	red	CSLC3 - cellulose synthase-like family C
LOC_Os08g30730	Tillers' number	8	18933746 .. 19119897	gray	holocarboxylase synthetase
LOC_Os08g15090	Root DW	8	9127576 .. 9198147	gray	diacylglycerol kinase catalytic domain family protein
LOC_Os08g36170	LBS	8	22548288 .. 23043330	gray	cytokinesis negative regulator RCP1
LOC_Os10g30156	Root Length	10	15344959 .. 15838006	gray	starch synthase
LOC_Os08g36760	LBS	8	22548288 .. 23401967	gray	remorin C-terminal domain containing protein
LOC_Os08g36140	LBS	8	22548288 .. 23043330	gray	expressed protein
LOC_Os05g01750	LBS	5	450809 .. 456689	gray	TruB family pseudouridylate synthase
LOC_Os08g36440	LBS	8	22548288 .. 23043330	gray	HVA22
LOC_Os08g30790	Tillers' number	8	18933746 .. 19119897	gray	hypersensitive-induced response protein
LOC_Os03g26260	LBS	3	14988188 .. 15124843	red	NB-ARC domain containing protein
LOC_Os06g40720	Tillers' number	6	24247418 .. 24286644	gray	EF hand family protein
LOC_Os10g28600	Root Length	10	14896094		PPR repeat domain containing protein
LOC_Os08g35760	LBS	8	22548288 .. 23043330	gray	Cupin domain containing protein
LOC_Os05g01530	LBS	5	340605 .. 430574	gray	expressed protein
LOC_Os10g29950	Root Length	10	15344959 .. 15838006	gray	speckle-type POZ protein
LOC_Os03g43810	Root Length	3	24533603 .. 24599472	gray	helix-loop-helix DNA-binding domain containing protein
LOC_Os08g40090	LBS	8	25320225 .. 25394695	gray	leucine rich repeat containing protein
LOC_Os08g36220	LBS	8	22548288 .. 23043330	gray	homeobox associated leucine zipper
LOC_Os05g01680	LBS	5	340605 .. 430574	gray	RCLEA7 - Root cap and Late embryogenesis related family protein precursor
LOC_Os10g30070	Root Length	10	15344959 .. 15838006	gray	sodium/calcium exchanger protein
LOC_Os05g13500	Root Length	5	7358986 .. 7554289	gray	expressed protein
LOC_Os03g44920	Root Length	3	25182239 .. 25467268	gray	OsFBX98 - F-box domain containing protein
LOC_Os03g57460	SBC Treated	3	32713371 .. 32877290	gray	fasciclin domain containing protein
LOC_Os10g29880	Root Length	10	15344959 .. 15838006	gray	expressed protein
LOC_Os05g01570	LBS	5	340605 .. 430574	gray	auxin-induced protein 5NG4
LOC_Os10g30180	Root Length	10	15344959 .. 15838006	gray	hsp20/alpha crystallin family protein
LOC_Os10g30200	Root Length	10	15344959 .. 15838006	gray	SKP1-like protein 1B
LOC_Os07g05550	SPAD	7	2593682	red	expressed protein
LOC_Os03g57490	SBC Treated	3	32713371 .. 32877290	gray	fasciclin domain containing protein
LOC_Os03g57600	SBC Treated	3	32713371 .. 32877290	gray	expressed protein
LOC_Os10g30310	Root Length	10	15344959 .. 15838006	gray	zinc finger, C3HC4 type domain containing protein
LOC_Os05g01600	LBS	5	340605 .. 430574	gray	actin
LOC_Os10g29650	Root Length	10	15344959 .. 15838006	gray	retrotransposon protein, putative, unclassified
LOC_Os07g05470	SPAD	7	2540573	red	AIR9 protein
LOC_Os03g19200	Root Length	3	10629354 .. 10850317	red	DNAJ heat shock N-terminal domain-containing protein
LOC_Os08g40080	LBS	8	25320225 .. 25394695	gray	expressed protein
LOC_Os03g11540	SBC Control	3	32713371 .. 32877290	gray	RPA1B - Putative single-stranded DNA binding complex subunit 1

P:red dot indicates -log *P*-value >4, gray, between 3 and 4 in MLM analysis

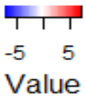

Supplementary Figure S4. Relative expression of 109 genes that were elected as candidate genes by GWAS and show a genotypic effect in transcriptional regulation. Values of relative expression in microarray hybridization: Treatment effect values means treated versus control samples, while genotype effect means sensitive versus tolerant genotype.

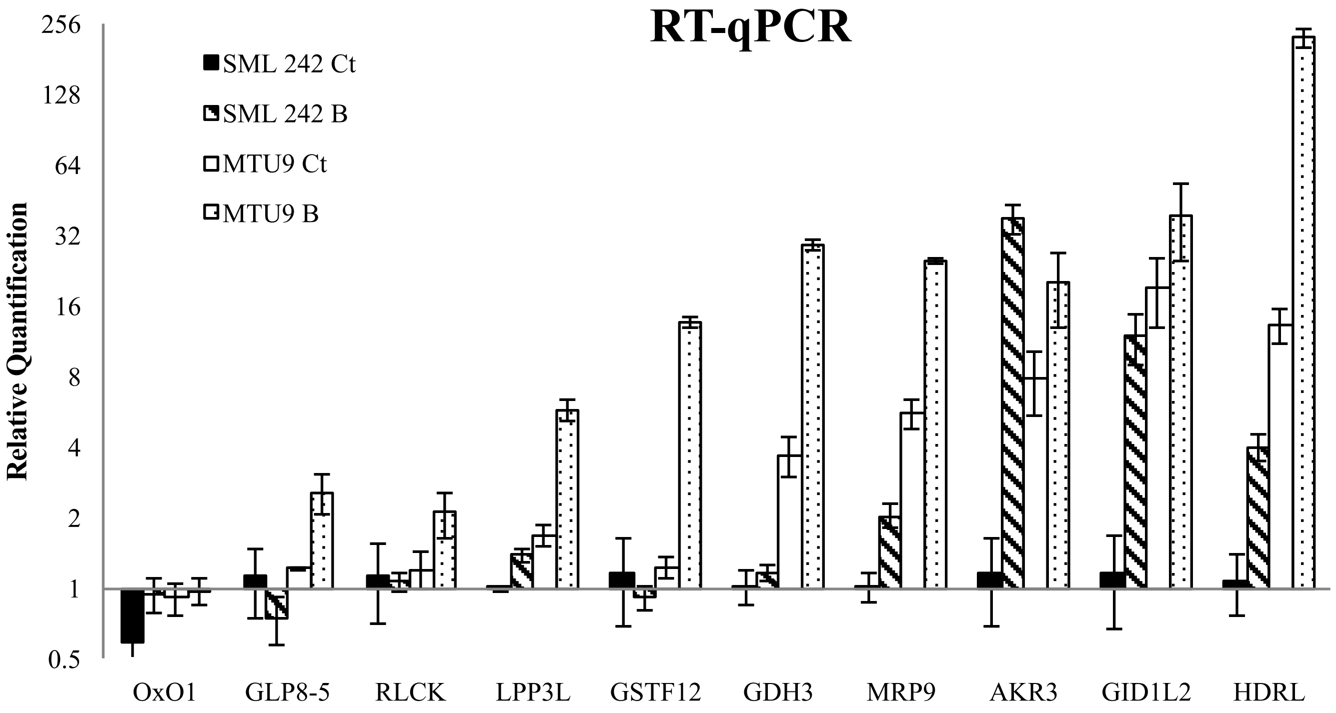

Supplementary Figure S5. Relative expression of ten rice genes used for validation of microarray data by RT-qPCR. Ox01(oxalate oxidase 1), GLP8-5(Germin-like protein 8-5), RLCK (Receptor-like Cytoplasmic Kinase 214), LPP3L(Lipid phosphate phosphatase 3- like), GSTF12(Phi Glutathione S-transferase 12), GDH3 (Glutamate Dehydrogenase 3), MRP9 (Multidrug Resistance-associated Protein 9), AKR3 (Aldo-Keto reductase 3), GID1L2 (gibberellin receptor), HDRL (4-hydroxy-3-methylbut-2-enyl diphosphate reductase-like). The U2 small nuclear ribonuclearprotein splicing factor (U2snRNP) was used as endogenous reference, and SML 242 in the control was used as the calibrator to express relative expression using the $\Delta\Delta C_T$ method.

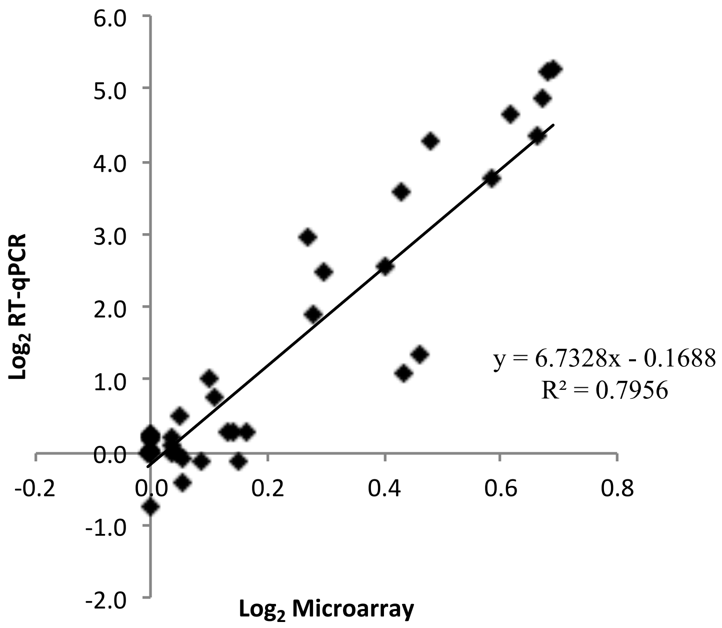

Supplementary Figure S6 . Validation of microarray data by RT-qPCR with ten selected genes. Data from both microarray and qRT-PCR were normalized by setting the expression level of SML 242 in the control as 1, and log to the base 2 of these relative expression levels were plotted. The primers for selected genes and complete expression patterns in microarray and qRT-PCR are given in Supplementary data S6.
